# Functional characterisation of Arabidopsis SPL7 conserved protein domains suggests novel regulatory mechanisms in the Cu deficiency response

**DOI:** 10.1186/s12870-014-0231-5

**Published:** 2014-08-30

**Authors:** Antoni Garcia-Molina, Shuping Xing, Peter Huijser

**Affiliations:** Department of Comparative Development and Genetics, Max Planck Institute for Plant Breeding Research, Cologne, 50829 Germany; Current address: Lehrstuhl für Systembiologie der Pflanzen, Technische Universität München, Emil-Ramann-Strasse 4, Freising, 85354 Germany; Current address: Department of Developmental Genetics, Centre for Plant Molecular Biology, Universität Tübingen, Auf der Morgenstelle 32, Tübingen, 72076 Germany

## Abstract

**Background:**

The Arabidopsis SQUAMOSA PROMOTER-BINDING PROTEIN-LIKE (SPL) transcription factor SPL7 reprograms cellular gene expression to adapt plant growth and cellular metabolism to copper (Cu) limited culture conditions. Plant cells require Cu to maintain essential processes, such as photosynthesis, scavenging reactive oxygen species, cell wall lignification and hormone sensing. More specifically, SPL7 activity promotes a high-affinity Cu-uptake system and optimizes Cu (re-)distribution to essential Cu-proteins by means of specific miRNAs targeting mRNA transcripts for those dispensable. However, the functional mechanism underlying SPL7 activation is still to be elucidated. As *SPL7* transcript levels are largely non-responsive to Cu availability, post-translational modification seems an obvious possibility. Previously, it was reported that the SPL7 SBP domain does not bind to DNA *in vitro* in the presence of Cu ions and that SPL7 interacts with a kin17 domain protein to raise SPL7-target gene expression upon Cu deprivation. Here we report how additional conserved SPL7 protein domains may contribute to the Cu deficiency response in Arabidopsis.

**Results:**

Cytological and biochemical approaches confirmed an operative transmembrane domain (TMD) and uncovered a dual localisation of SPL7 between the nucleus and an endomembrane system, most likely the endoplasmic reticulum (ER). This new perspective unveiled a possible link between Cu deficit and ER stress, a metabolic dysfunction found capable of inducing SPL7 targets in an SPL7-dependent manner. Moreover, *in vivo* protein-protein interaction assays revealed that SPL7 is able to homodimerize, probably mediated by the IRPGC domain. These observations, in combination with the constitutive activation of SPL7 targets, when ectopically expressing the N-terminal part of SPL7 including the SBP domain, shed some light on the mechanisms governing SPL7 function.

**Conclusions:**

Here, we propose a revised model of SPL7 activation and regulation. According to our results, SPL7 would be initially located to endomembranes and activated during ER stress as a result of Cu deficiency. Furthermore, we added the SPL7 dimerization in the presence of Cu ions as an additional regulatory mechanism to modulate the Cu deficiency response.

**Electronic supplementary material:**

The online version of this article (doi:10.1186/s12870-014-0231-5) contains supplementary material, which is available to authorized users.

## Background

SQUAMOSA PROMOTER BINDING PROTEINS (SBP) constitute a transcription factor (TF) family exclusively found in green plants. *Arabidopsis thaliana* (hereinafter Arabidopsis) homologs have been related to developmental and adaptive programmes, such as plastochron determination [1], leaf morphogenesis [2], vegetative phase transition [3], flowering [4], anther and gynoecium development [5–7] or innate immunity [8] and copper deficiency response [9,10].

Despite evolutionary divergence between the different family members, the tertiary structure of all SBP proteins encompasses the founding SBP-domain. It consists of a 76 amino acid signature including a functional bipartite nuclear localisation signal (NLS) and a series of 8 conserved cysteine and histidine residues organized in two unconventional zinc fingers (ZF1 and ZF2) [11–13]. Structural and functional studies suggested that ZF1 would maintain the SBP folding, while ZF2 would confer selectivity for the DNA sequence to bind [14,15]. Therefore, the SBP domain facilitates nuclear translocation and confers the capability to bind DNA-motifs harbouring a GTAC core sequence [11,16,17].

The *SBP* genes appear in moderately sized-families. The Arabidopsis genome encodes 16 different SBP-Like (SPL) proteins grouped in 2 classes according to size, sequence similarity and structure and expression patterns of the respective genes. Based on these criteria, the denoted large *SPLs* (*SPL1/7/12/14/16*) conform a class representing the most complex and constitutively expressed genes. The other class is constituted by the small *SPLs*, whose expression is refined by the well-conserved and related microRNAs *miR156/7*, with *SPL8* as a notable exception [18,19].

In recent years, the *Chlamydomonas reinhardtii* Copper response regulator Crr1 and its closest Arabidopsis homolog SPL7 attracted attention because of their deeply conserved function as central orchestrators of Cu homeostasis [9,10,17]. Cu is an essential micronutrient for virtually all eukaryotes since its redox properties are optimal for essential catalytic functions in protein complexes. Indeed, plant cells rely on Cu-proteins to deal with oxidative stress, energy production, lignification, or pollen tube guidance [20,21]. Furthermore, Cu has also been reported to play a structural role in the ethylene and salicylic acid receptors, as well as in the molybdenum cofactor [22–24]. However, an excess of free Cu ions will damage cellular components, e.g. lipids, proteins or nucleic acids, due to the generation of reactive oxygen species (ROS) [25]. To cope with this dual nature of Cu, cells possess a fine-tuned homeostatic network aimed at maintaining Cu levels within a proper range. Although the general features of this network are conserved among all eukaryotes, main evolutionary divergences concern the regulatory mechanisms. During Cu starvation in Arabidopsis, SPL7 directly binds to GTAC motif-containing Cu response elements (CuRE) located in the promoter regions of Cu responsive genes [10,11]. In a first response, a Cu-uptake system based on the Cu-metalloreductases FRO4/5 and the plasma membrane-related Cu transport proteins COPT1/2/6 is promoted [9,10]. Secondly, SPL7 reprograms cellular gene expression for a more efficient Cu usage and (re-)distribution within the plant, thereby prioritizing delivery to essential enzymes. In this way, levels of particular microRNAs, denoted Cu-miRNAs and including *miR398* and *miR408*, are raised to translationally repress production of non-essential Cu-requiring proteins, such as the cytosolic Cu/Zn superoxide dismutase (CSD1), chloroplastic CSD2, plantacyanin or the laccases. Suppression of CSD2 and the promotion of FSD1 represent a coordinated substitution of the chloroplastic superoxide dismutases that facilitates a preferential delivery of Cu to plastocyanin (PC) [9,10,20].

However, the mechanism underlying SPL7 activation is not fully understood, especially with regard of Cu sensing and protein regulation. *SPL7* is a constitutively expressed gene detected in all plant tissues regardless of Cu availability. Consequently, a post-translational regulation for this TF has been proposed [9,10,20]. Within this context, we recently reported the physical interaction between SPL7 and a kin17-domain encoding protein (KIN17) to stimulate SPL7 targets during Cu starvation [26]. Moreover, the *in vitro* SBP-DNA binding could be prevented by Cu ions probably replacing complexed Zn ions and thereby changing the conformation of SPL7-like proteins [14,17]. Here, we present a functional characterisation of conserved domains in the SPL7 protein as to come to a better understanding of how its activity may be regulated in response to cellular Cu status in Arabidopsis. Our subcellular and biochemical approaches revealed that the presence of a TMD recruits SPL7 to the microsomal fraction, likely at the ER membrane and suggests a proteolytic cleavage prior to its nuclear translocation. Interestingly, our data indicate that Cu deficiency implicates ER stress and could constitute a driving force to activate SPL7. Moreover, a SPL7 dimerization domain could act in a mechanism to prevent the protein from entering the nucleus.

## Results

### A conserved transmembrane domain is sufficient to anchor SPL7-like proteins to the plasma membrane

**Figure 1:**
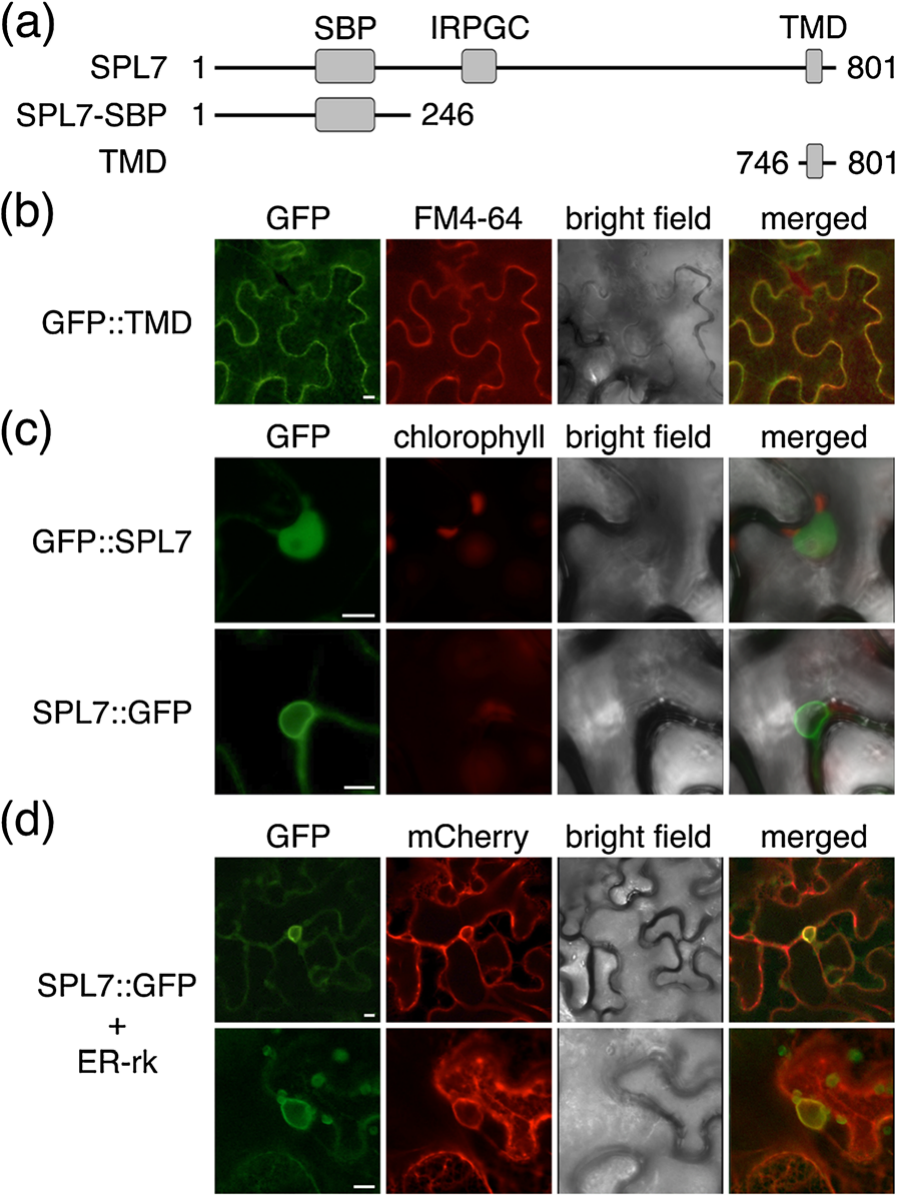
**Subcellular localization of GFP-tagged SPL7 protein derivatives transiently expressed in tobacco leaf-epidermal cells. (a)** Graphic depiction of SPL7 protein derived polypeptides used in this work. The conserved domains (SBP; IRPGC; TMD) are indicated with grey boxes. The position of the amino- and carboxy-terminal amino acid residues relative to the full size SPL7 protein is provided. **(b)** Confocal microscopy reveales co-localization of a translational fusion between the predicted SPL7 transmembrane domain and GFP (GFP::TMD) with the plasma membrane marked with the styryl dye FM4-64 **(c)** Expression of the entire SPL7 coding sequence fused in frame to GFP either at the amino- or carboxi-terminal ends (GFP::SPL7 and SPL7::GFP) results in a dual localization within or around the nucleus, respectively. **(d)** The carboxi-terminal GFP-tagged SPL7 (SPL7::GFP) co-localizes with the endoplasmic reticulum marked through co-infiltration with an mCherry-tagged ER marker (ER-rk). In all cases, representative images of the GFP, FM4-64, chlorophyll and mCherry signals are shown together with the corresponding bright field and merged images. Scale bars, 10 μm in **(b-d)**.

In order to identify conserved protein signatures possibly participating in SPL7 function, we carried out a comprehensive multiple alignment among SPL7 and orthologs from different species including di- and monocots, a gymnosperm, a bryophyte and green algae. Initially, we paid attention to a 20 amino acid hydrophobic region located in the carboxy-terminal region, and found it to be conserved in all higher plant SPL7 homologs (Additional file 1: Figure S1a). The TMHMM prediction service (www.cbs.dtu.dk/services/TMHMM/ [27]) retrieved this region as a putative TMD. Therefore, to investigate whether this domain is capable of tethering SPL7 to cellular membranes, we fused the TMD C-terminal to the green fluorescent protein (GFP) (GFP::TMD; Figure 1a) and transiently expressed it in tobacco leaves. In order to discriminate the plasma membrane, samples were incubated with the lipophilic styryl dye FM4-64 under cold conditions. As shown in Figure 1b, confocal imaging revealed a GFP signal outlining the transformed cells and perfectly overlapping with the FM4-64 signal.

**Figure 2:**
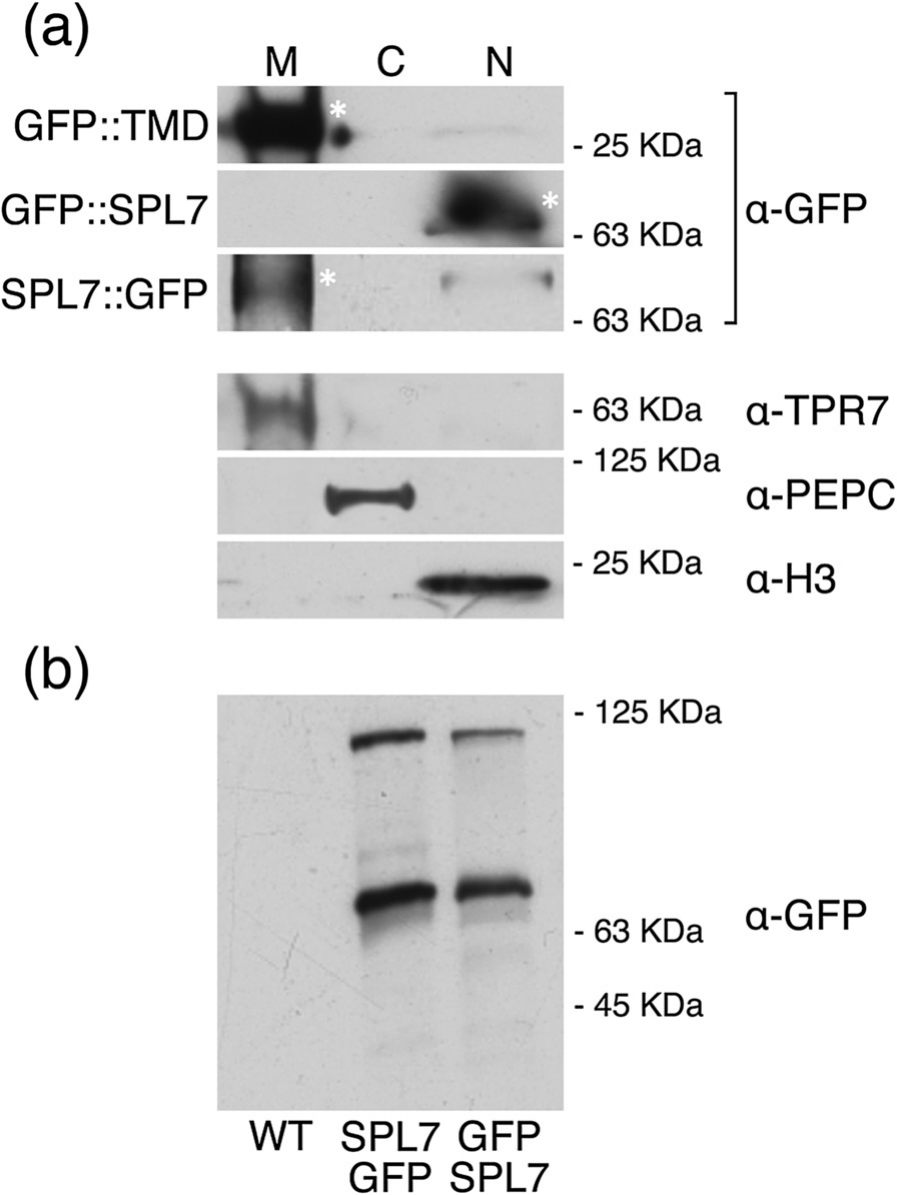
**Biochemical analysis of SPL7 subcellular localization and processing. (a)** Total protein extracts from tobacco leaves transiently expressing different GFP-tagged SPL7 versions were subjected to biochemical fractionation, as described in Experimental Procedures and analysed by Western blotting with antibodies against GFP (α-GFP). Antibodies against the organelle markers TPR7 (α-TPR7), PEPC (α-PEPC) and histone H3 (α-H3) were used to validate the fractionation. M, microsomes; C, cytosol; N, nucleus. Main bands are highlighted with an asterisk. **(b)** Proteolytic processing of transiently expressed GFP::SPL7 and SPL7::GFP by Western blotting. WT, protein extract of non-transformed tobacco leaves. Sizes of molecular-weight markers run in the same gels are shown alongside the blots in **(a-b)** according to manufacturer’s indications for 10% SDS-PAGE gels.

To further confirm our data, we also performed a biochemical fractionation using total extracts from transfected tobacco leaves. Enriched microsomal (M), cytosolic (C) and nuclear (N) fractions were analysed by Western blot using antibodies against GFP and selected organelle markers. In this case, the GFP::TMD clearly associated with the microsomal fraction (Figure 2a). Altogether these observations point to the predicted TMD domain as able and sufficient to anchor proteins to the plasma membrane.

### SPL7 exhibits a dual subcellular localisation and likely requires proteolytic cleavage to become translocated to the nucleus

The above-stated results concerning the presence of a TMD seem to oppose the function of a conserved bipartite NLS within the SBP domain and the rather constitutive nuclear localization reported in our previous observations, as well as for several other SBP-domain proteins [11,14,26]. Therefore, we addressed the question whether SPL7 could display a dual subcellular distribution. For this purpose, we generated CaMV 35S promoter-driven transgenes consisting of the entire *SPL7* coding region and fused in frame either 5′ or 3′ to GFP to allow the constitutive expression of either N-terminal or C-terminal tagged SPL7 protein (GFP::SPL7 and SPL7::GFP; Figure 1a). Since we failed to reliably detect GFP fluorescent signals in Arabidopsis plants stably transformed with these constructs, we decided to use agro-infiltration of tobacco leaves as a heterologous system to assess the subcellular localization of the encoded protein products. Strikingly, while GFP::SPL7 distributed homogeneously within the nucleoplasma excluding the nucleolus, the C-terminal tagged version located around the nucleus and to filamentous structures in cytoplasmic strands (Figure 1b, 1c and Additional file 2: Figure S2). As the latter pattern suggested possible association with the ER, we co-infiltrated the SPL7::GFP-encoding construct with an ER marker fused to the mCherry fluorescent protein (ER-rk [28]). This revealed a high degree of co-localization of both fluorescent signals, most intensely around the nucleus (Figure 1d). Moreover, we also subjected total extracts from *35S::GFP::SPL7* and *35S::SPL7::GFP* transformed leaves to a biochemical fractionation, as described above. Indeed, GFP::SPL7 protein was detected in the nuclear enriched fraction, whereas SPL7::GFP primarily associated with the microsomal fraction (Figure 2a), thereby corroborating the microscopic observations. Consequently, these data strongly suggest SPL7 to distribute between the nucleus and the endomembrane system.

Interestingly, although estimating that GFP would contribute ~23 KDa and SPL7 ~ 90 KDa, the observed apparent molecular weight of both GFP-tagged SPL7 versions seemed more consistent with ~63 KDa (Figure 2a). Since membrane-anchored proteins must be somehow released prior to their translocation to the nucleus and exert their function, we investigated if these observed bands could correspond to cleaved SPL7 products. To this end, total protein extracts from transformed tobacco leaves expressing either GFP::SPL7 or SPL7::GFP were also analysed by Western blot. A pattern including two specific bands was obtained regardless of the position of the tag (Figure 2b). We considered that the upper band (~125 KDa) could correspond to the full-sized SPL7 while the presence of the second lower band (~63 KDa) in both cases might be explained if SPL7 would have been cleaved in the middle (Figure 2a). This processing would thus render a derived polypeptide fitting the observed size (~45 KDa from half of SPL7 + ~23 KDa from GFP; Figure 2a,b).

These results are consistent with an arranged pattern where the N-terminal half of SPL7 translocates to the nucleus following proteolytic cleavage, whereas the C-terminal half would remain attached to some endomembrane, such as the ER.

### Cu deficiency generates endoplasmic reticulum stress, a metabolic perturbation that promotes SPL7 target activity

**Figure 3:**
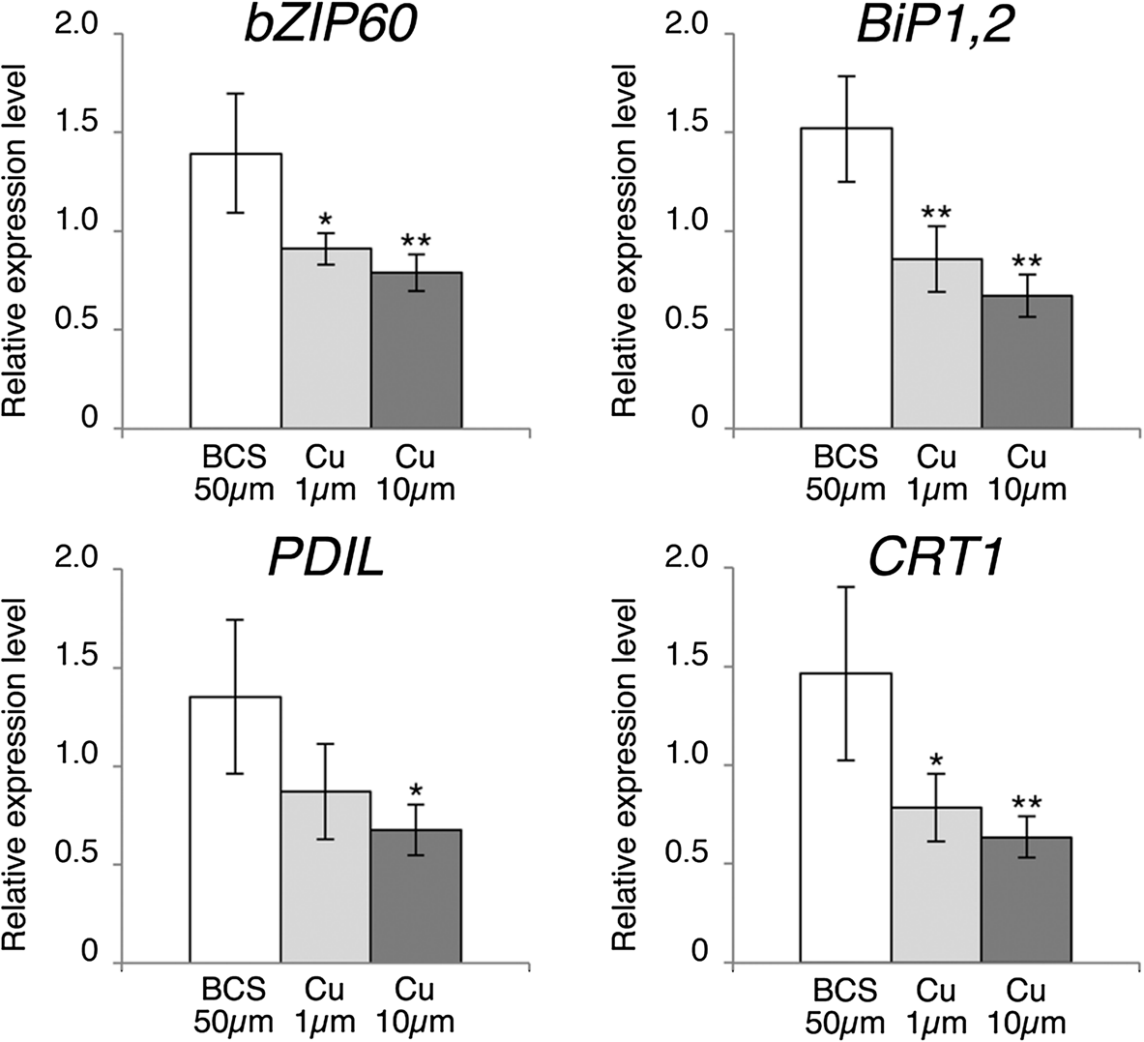
**Cu deficiency induced ER-stress markers.** The relative mRNA levels of indicated ER-stress markers were determined by qPCR on RNA from 7-day-old wild-type Arabidopsis seedlings grown on ½ MS supplemented with BCS 50 μM, CuSO_4_ 1 μM or 10 μM. Error bars indicate standard deviation (n ≥ 3 independent biological samples), asterisks indicates statistically significant difference to Cu deficiency conditions in Student’s *t*-test (* p < 0.05; ** p < 0.01).

Because *SPL7* transcript levels remain relatively constant irrespective of Cu availability, post-translational mechanisms have been proposed to regulate this TF [9,10,20,26]. Assuming that SPL7 could be initially attached to the ER membrane, we wondered whether perturbations affecting the functionality of this organelle would trigger SPL7 processing and activation. In this context, it is well known that adverse environmental conditions result in miss-folding of ER-resident proteins [29,30]. To counteract this so-called ER stress, a defined unfolded protein response (UPR) is generated through the activation of genes coding for folding-assisting proteins [29,30]. Curiously, genes categorized as UPR markers appeared relatively down-regulated in a transcriptomic assay in shoots from plants hydroponically cultured on Cu sufficient media [9]. Thus, we decided to investigate whether varying Cu supply may influence ER stress. Thereto, transcript levels of reported UPR markers were determined in seedlings grown on ½ MS media supplemented with either the Cu-specific chelator BCS 50 μM (Cu deficiency), CuSO_4_ 1 μM (Cu sufficiency) or 10 μM (Cu excess). Interestingly, our selected markers, including the TF bZIP60 (At1g42990), the disulfide isomerase-like protein (PDIL; At1g21750), the luminal binding proteins BiP1,2 (At5g28540/At5g42020) and calreticulin (CRT1; At1g56340) were all slightly induced (ca. 1.4-fold) following Cu deficient conditions (Figure 3). Our results thus uncovered that Cu deficiency to some degree seems to result in ER stress.

**Figure 4:**
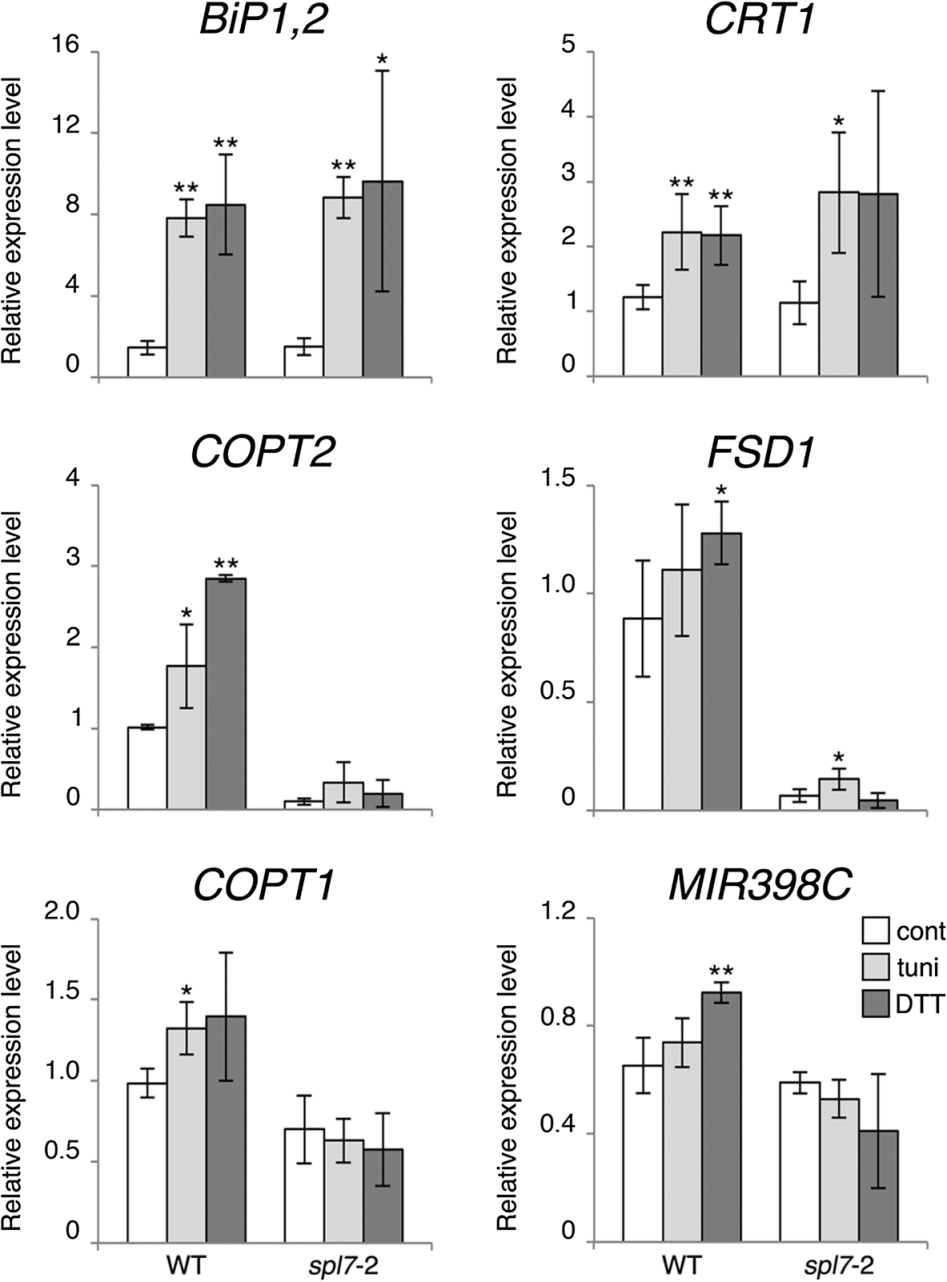
**Induction of SPL7 targets following ER stress.** Arabidopsis wild-type (WT) and *spl7*-2 mutant seedlings grown on ½ MS supplemented with CuSO_4_ 0.5 μM for 5 days were transferred to liquid ½ MS (control; cont) or to liquid ½ MS supplemented with tunicamycin (5 μg/mL; tuni) or DTT (2 mM; DTT) for 3 hours. Total RNA was isolated and relative transcript levels of selected SPL7 targets monitored by qPCR. Error bars indicate standard deviation (n ≥ 3 independent biological samples), asterisks statistically significant difference to control conditions in Student’s *t*-test (* p < 0.05; ** p < 0.01).

To further investigate the likely connection between ER stress and SPL7 activation, we monitored the behaviour of SPL7 target genes in seedlings treated with UPR-inducing drugs. Thereto, 5-day-old wild type seedlings grown on low but Cu sufficient medium (½ MS supplemented with CuSO_4_ 0.5 μM [31]) were incubated for 3 h on liquid ½ MS with tunicamycin [32], an inhibitor of N-linked protein glycosylation, or dithiothreitol (DTT), disrupting disulphide bond formation. The presence of ER stress in our experimental conditions was confirmed by raised *BiP1,2* and *CRT1* transcript levels in comparison to controls (Figure 4). Moreover, the transcript abundance of the analysed SPL7 targets was generally increased, with DTT producing a more prominent effect (Figure 4). Indeed, seedlings exposed to DTT raised transcript levels of *FSD1*, *COPT1* and *MIR398C* ca. 1.4-fold in comparison to untreated controls, while *COPT2* showed the strongest induction (2.8-fold) (Figure 4). In addition, since this response could not be observed in the *spl7*-2 mutant line, we concluded that the stimulation of the Cu-response during ER stress is SPL7-dependent. Altogether, our results suggest that Cu deficiency induces ER stress, which could be used as a signal to promote the activation of SPL7.

### SPL7 is able to homodimerize *in vivo*

**Figure 5:**
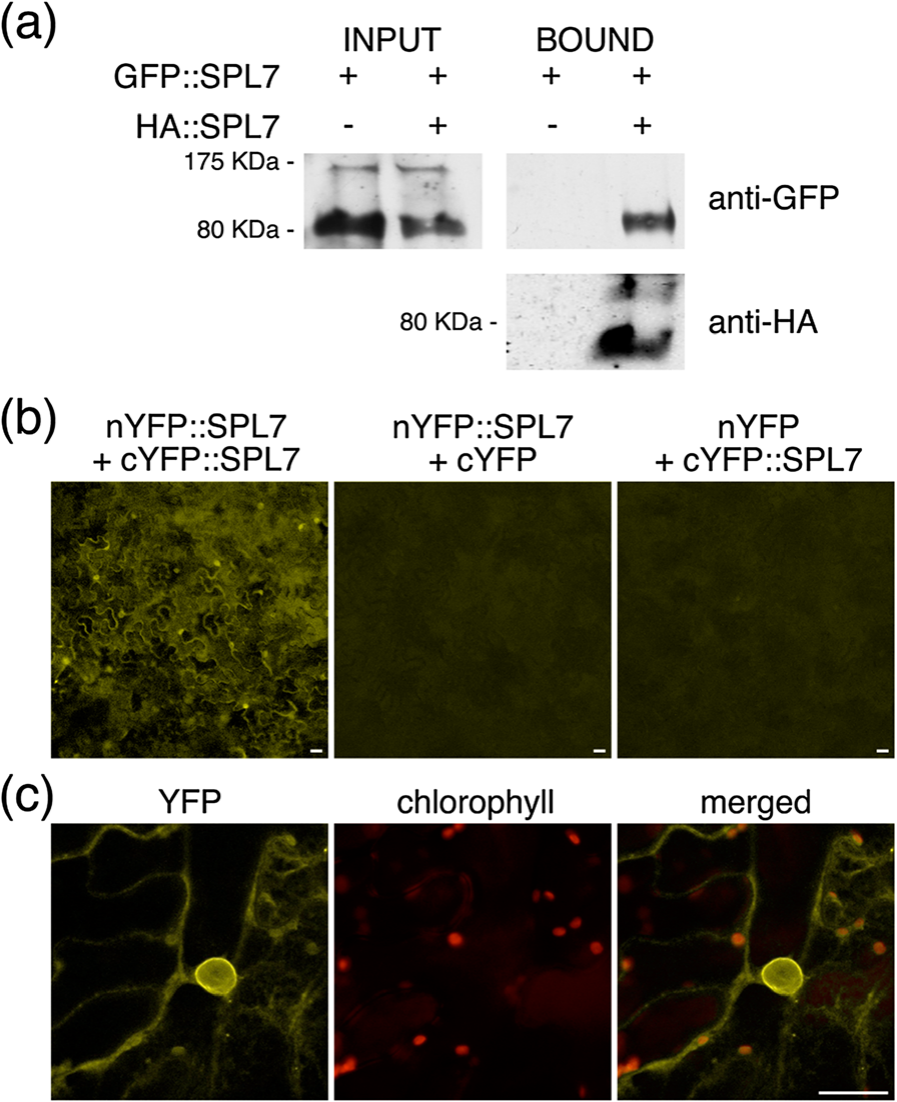
**SPL7 homodimerizes** ***in vivo*** **. (a)** For a pull-down assay by means of an antibody against HA (BOUND), total protein was extracted from tobacco leaves transiently expressing full-size SPL7 tagged either with GFP or HA epitopes (GFP::SPL7 and HA::SPL7; INPUT). Input and bound fractions were assayed by Western blot using an anti-GFP antibody to assess GFP::SPL7 co-immunoprecipitation. Membranes were reprobed with an anti-HA-HRP (anti-HA) antibody to check for HA::SPL7 pull-down. Sizes of molecular-weight markers run on the same gels are indicated at the left according to manufacturer’s indications for precasted gels. Note that the apparant molecular weights may differ in comparison to those shown in Figure 2 due to the use of a different separation matrix for electrophoresis. **(b)** For bimolecular fluorescent complementation (BiFC) analysis the split-YFP tags were N-terminal fused to the full-size SPL7 protein (nYFP::SPL7, cYFP::SPL7) and co-expressed in tobacco leaves. Restoration of the YFP fluorescence signal could be observed with widefield epifluorescence microscopy using a YFP band-pass filter. Co-expression of the individual constructs with their complementary empty vectors (middle and right panels) did not result in reconstitution of YFP fluorescence. **(c)** A representative confocal microscopic image of the reconstituted YFP fluorescence illustrating its predominant extranuclear localization is shown together with chlorophyll autofluorescence and merged images. Scale bars, 25 μm.

Protein-protein interactions could also constitute a post-translational mechanism to refine SPL7 function. Therefore, we conducted a yeast-two-hybrid (Y2H) screening aimed at identifying putative SPL7-interacting proteins. Besides KIN17, on which we recently reported [26], 8 different preys corresponding to SPL7 itself were fished when using an SPL7 fragment as bait, strongly suggesting SPL7 homodimerization (Additional file 3: Figure S3). Interestingly, all preys encompassed an evolutionary well-conserved ca. 50 aa signature marked by the so-called IRPGC domain (Figure 1a, Additional file 1: Figure S1 and Additional file 3: Figure S3 [16,17]). To further confirm the SPL7-SPL7 interaction, the entire SPL7 coding sequence was amino-terminally fused to the *Influenza* hemagglutinin (HA) epitope tag (HA::SPL7) and co-expressed with GFP::SPL7 in tobacco leaves. Subsequent Western blot analysis uncovered that GFP::SPL7 co-immunoprecipitated with HA::SPL7 in a HA pull-down assay (Figure 5a). Remarkably, because the co-immunoprecipitated peptides corresponded in size to the putative SPL7-processed version (Figure 5a), we concluded that the dimerization likely constitutes a post-cleavage event. Moreover, the SPL7 homodimerization was also ascertained by bimolecular fluorescence complementation (BiFC). For this purpose, in-phase translational fusions between the entire SPL7 coding sequence and the two split-yellow fluorescent protein (YFP) halves in amino-terminal position (nYFP::SPL7 and cYFP::SPL7) were generated and co-expressed in tobacco leaves. Whereas expression of the individual constructs with the complementary empty vector did not generate any specific YFP-derived fluorescence, concomitant expression resulted in a YFP signal mainly located at the nuclei in widefield epifluorescence microscopy (Figure 5b). However, confocal microscopy enabled a more accurate observation of the reconstituted YFP fluorescence signal and revealed a pattern mirroring the endomembrane system, as previously observed for SPL7::GFP, and largely excluded from the nucleus (Figures 1c and 5c). On the contrary, a YFP signal could not be reconstituted when using carboxy-terminal fusions (data not shown). Taken these data together, we conclude that SPL7 dimerization takes place outside the nucleus, probably at or in the vicinity of the ER after being processed. We may envisage that this dimerization constitutes a regulatory mechanism to restrict SPL7 from entering the nucleus, i.e. as a negative feedback mechanism.

### Arabidopsis transgenic lines expressing the SPL7-SBP domain exhibit constitutive activation of SPL7 targets

**Figure 6:**
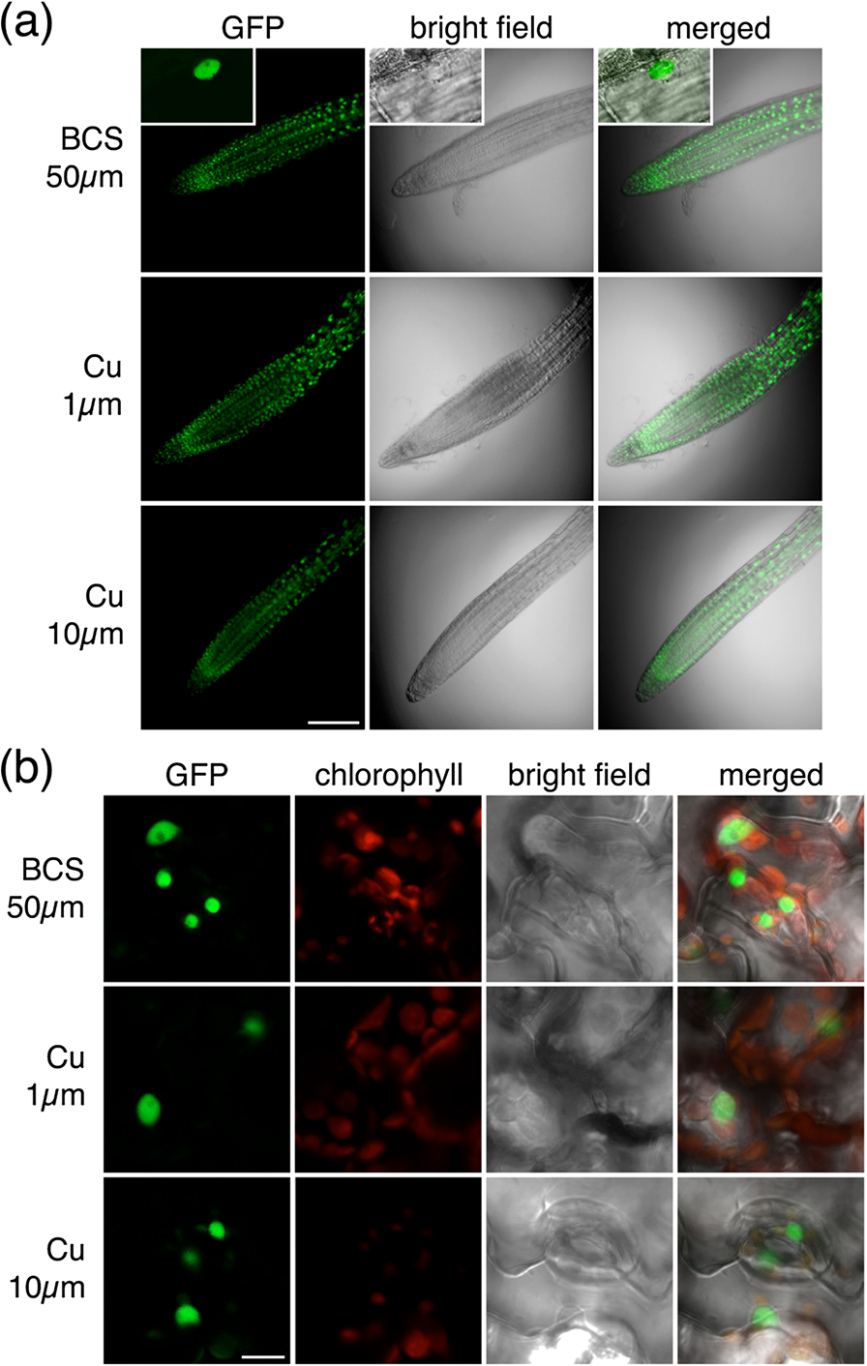
**An** ***SPL7-SBP*** **transgene results in constitutive detectable levels of nuclear localized protein in stably transformed Arabidopsis.** Seven-day-old *spl7*-2 mutant seedlings expressing the GFP-tagged N-terminal part of SPL7, including the SBP-domain but excluding the presumed dimerization domain, were grown on ½ MS supplemented with BCS 50 μM, CuSO_4_ 1 μM or 10 μM. Roots **(a)** and leaves **(b)** were examined using confocal microscopy. The insets in the upper row of panel **(a)** show a fluorescent nucleus at a higher magnification. GFP and chlorophyll fluorescence is provided together with bright field and merged images. Scale bars, 100 μm **(a)** and 10 μm **(b)**.

In addition to the above-mentioned post-translational processing and protein-protein interactions, i.e. proteolytic cleavage and dimerization, SPL7 function may also be altered following conformational changes. Indeed, Cu ions have been reported to preclude both Chlamydomonas Crr1-SBP and SPL7-SBP DNA-binding capability *in vitro* [14]. Consequently, the replacement of Zn ions by Cu at the ZFs results in a conformational change that could constitute an additional regulatory mechanism to inactivate SPL7. To further validate this postulate *in vivo*, stably transformed lines constitutively expressing a GFP-tagged SPL7 protein truncated immediately behind the SBP domain were generated in the *spl7-*2 background (GFP::SBP; Figure 1a). Importantly, GFP::SBP/*spl7*-2 lines were not only able to complement the *spl7* mutant phenotypes under Cu limitation with respect to root growth, size or silique production (Additional file 4: Figure S4), but a GFP signal also became microscopically detectable *in planta* (Figure 6). GFP::SBP fluorescent signal could be detected at high levels within nuclei of both roots and shoots of 5-day-old seedlings grown on media differing in Cu availability (Figure 6). These observations indicate that Cu availability does not markedly affect stability of the SPL7-SBP domain.

**Figure 7:**
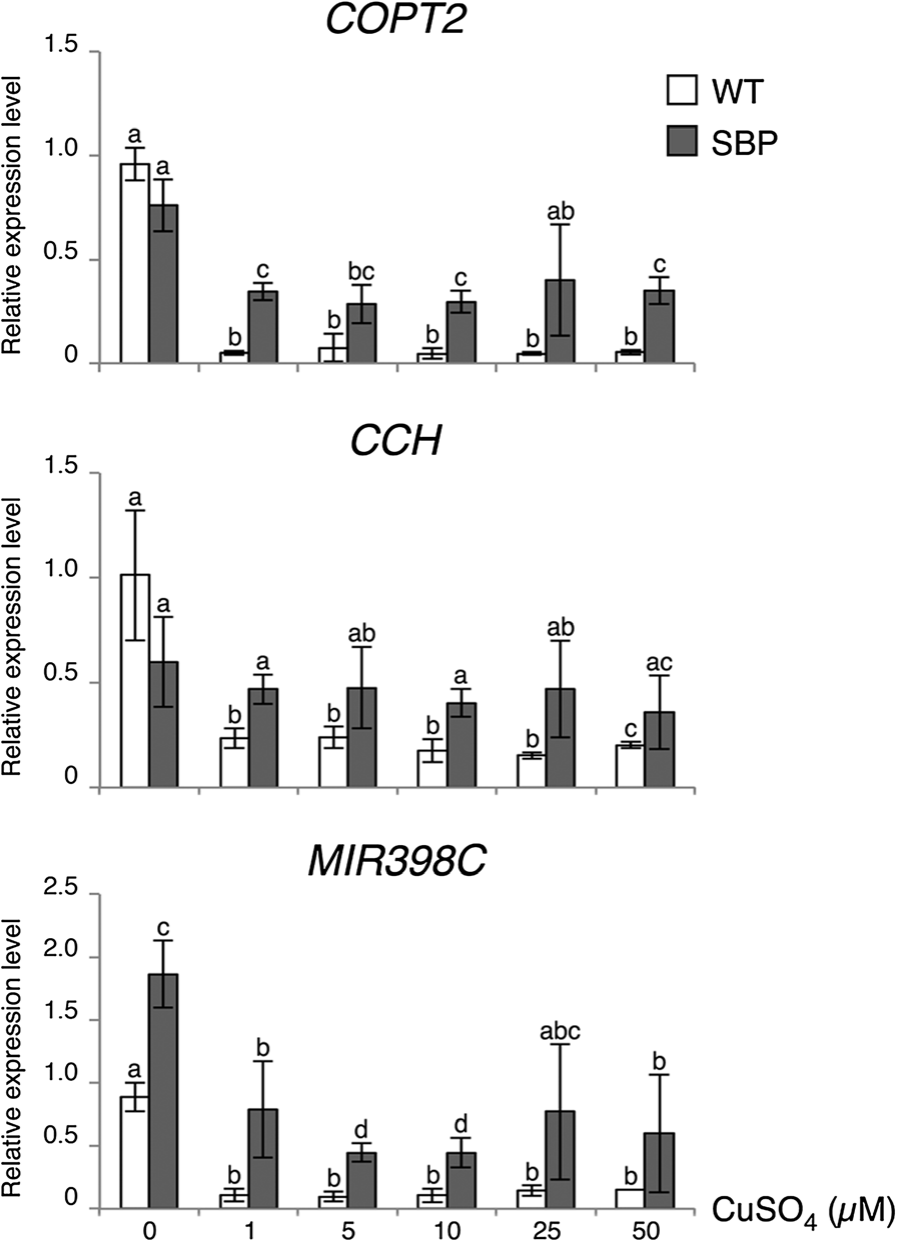
**SPL7-SBP promotes constitutive expression of SPL7 targets in stably transformed Arabidopsis.** mRNA levels of the genes indicated were determined by qPCR on total RNA from 7-day-old wild-type and *spl7*-2 mutant lines grown on ½ MS supplemented with CuSO_4_ (0 to 50 μM) and constitutively expressing GFP-tagged SPL7-SBP. Error bars indicate standard deviation (n ≥ 3 independent biological samples) and letters statistically significant differences among samples in Student’s *t*-test (p < 0.05).

Then, to learn if Cu actually impedes functionality of the SBP domain, transcript levels of selected SPL7-targets were monitored by qPCR in 7-day-old wild-type and GFP::SBP/*spl7*-2 seedlings grown on a gradient of Cu concentrations. To this end, the standard ½ MS (Cu deficiency) was supplemented with CuSO_4_ to achieve Cu sufficiency (1 μM) or Cu excess (5–50 μM). As shown in Figure 7, *COPT2*, *pre-miR398c* and *CCH* transcript levels in wild type already reached their minimum in the presence of 1 μM Cu, i.e. a drastic reduction of ca. 20-fold, 10-fold and 4-fold, respectively, compared to Cu deficient conditions. In contrast, a similar comparison between the GFP::SBP/*spl7*-2 lines showed a more moderate reduction of ca. 5-fold for *CCH* and less than 2-fold for *COPT2* and *MIR398C* and remained significantly higher in comparison to wild type (Figure 7). Moreover, these elevated levels in the transgenic seedlings remained largely constant along the gradient, even under physiologically incompatible Cu conditions (Figure 7).

All together, our data indicate that neither the stability of the SBP domain nor its function is severely affected by Cu ions *in planta*. Based on these results, we suggest that protein domains outside the SBP-domain of SPL7 are likely to have a more profound effect on SPL7 activity in response to Cu availability.

## Discussion

**Figure 8:**
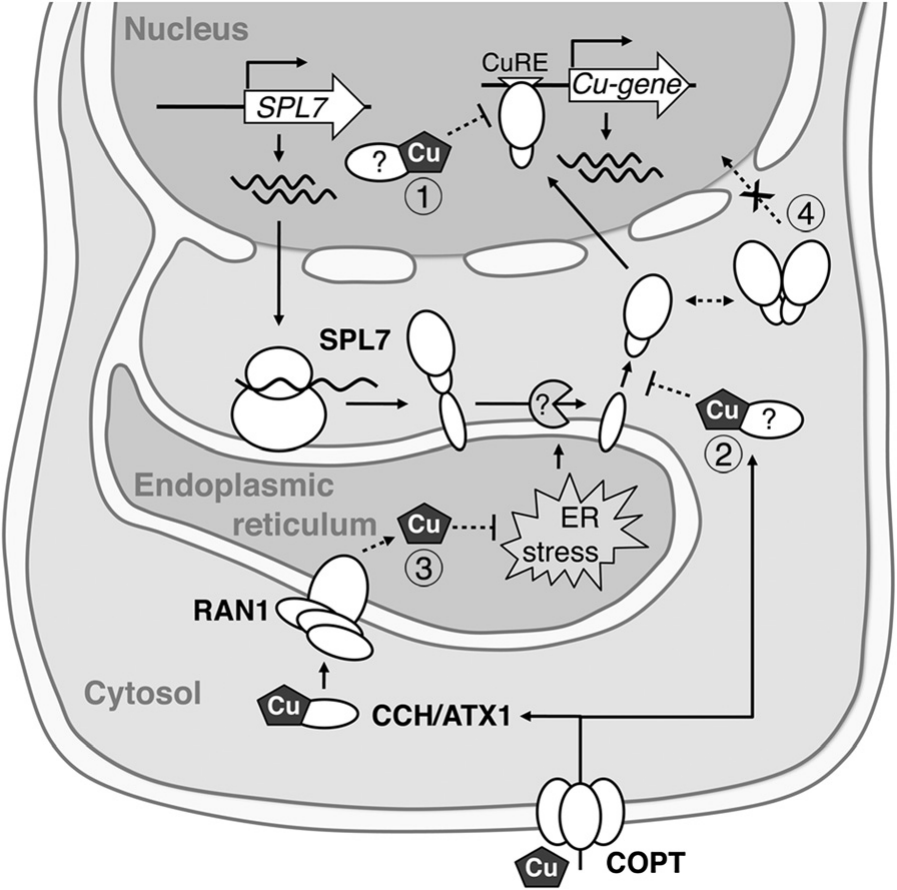
**A working model for the regulation of SPL7 function.** Cu supply depends on extracellular input and mobilization from intracellular stores via selective Cu-transport proteins, COPT. Acquired Cu is complexed by a set of metallochaperones, like CCH and ATX1, and delivered to specific targets. As main orchestrator of the Cu starvation response, SPL7 may be among these targets in order to become or remain repressed in the presence of sufficient Cu. This may be achieved through a direct interaction with delivered Cu resulting in an inability of SPL7 to bind to CuRE motifs in the promoter regions of its targets genes (1). In addition or alternatively, proteolytic processing of ER membrane-tethered SPL7 may be repressed in the presence of Cu (2). As a consequence, activation of SPL7 in response to Cu-deficiency may thus result from a relief of these repression mechanisms. Furthermore, ER stress caused by a failure to fulfil the demand for Cu of proteins involved in the secretory pathway, may actively promote the release of the membrane-bound SPL7 (3). Finally, a precocious dimerization to modulate the Cu deficiency response as the amount of released SPL7 continues to raise may prevent SPL7 from entering the nucleus either due to NLS masking or exceeding the size exclusion limit of the nuclear pore (4).

Green plants, from single-celled algae to angiosperms, rely on an evolutionary well-conserved SBP-box TF to orchestrate their adaptive response to Cu deprived periods. As potential TFs, all SPL7-like proteins contain a functional bipartite NLS overlapping ZF2 within the SBP-domain [11,12,26]. However, our cellular and biochemical approaches confirmed the anchoring of SPL7 to the microsomal fraction, most likely to the ER-membrane, through a C-terminal TMD (Figure 8). Consequently, a not yet identified molecular mechanism must facilitate the observed dual localization of SPL7 in cells.

In this sense, ER-membrane tethered TFs (ER-MTTFs) might provide an illustrative example to infer the SPL7 mechanism since they exhibit a similar behaviour. This class of TFs display an initial latent form when attached to membranes and require some sort of processing to be released and eventually translocated to the nucleus [33]. ER-MTTFs nuclear-localised versions are generated as a result of two main strategies, namely mRNA processing and proteolytic cleavage. Although alternative mRNA splicing has been reported to produce a non-anchored version of the bZIP60 ER stress transducer [34,35], this mechanism would not be expected for SPL7 because its known or predicted splicing variants (AT5G18830.2 and -.3; TAIR10 genome release; www.arabidopsis.org) do not disrupt the TMD domain. More often, specific proteolytic activities, such as the regulated intramembrane proteolysis (RIP) and the rhomboid proteases, produce a cleavage at the vicinity of the TMD [36,37]. However, because the apparent molecular weights of both SPL7 nuclear and ER-attached fragments correspond approximately to half of that of the predicted full-size protein, a proteolytic cleavage in the middle is suggested as the strategy to release and activate SPL7 (Figure 8). Thus, regulated ubiquitin/proteasome-dependent processing (RUP) and the so-called receptor-activated proteolysis (RAP) would be more conceivable for this case [38,39]. Nevertheless, we envisage a relative rapid-acting mechanism as the presence of the full-sized GFP::SPL7 was barely detectable in co-immunoprecipitation experiments and remained even undetectable following biochemical fractionation. Consequently, it would be particularly interesting to identify the responsible protease(s) and the cleavage site(s) in SPL7, as it will shed more light on the precise mechanism activating SPL7 and contribute to relate its function to additional biological responses.

The initial location of a likely dormant SPL7 at the ER provides a new perspective on the regulation of Cu homeostasis and requires a re-evaluation of the role of the so-called secretory pathway in Cu sensing. As represented in Figure 8, Cu^+^ imported by the selective Cu-transport proteins CTR/COPT is bound and further distributed by Cu-specific soluble factors or metallochaperones (for a comprehensive description see Burkhead and collaborators [20]). Among them, ATX1-like metallochaperones interact with the P_B_-ATPase Ccc2 in *Saccharomyces cereviseae*, or RAN1/HMA7 in plants, in order to supply Cu-proteins *en route* [40,41]. Whereas Ccc2 resides in the Golgi apparatus of yeast, the exact subcellular localization of Arabidopsis RAN1 has not yet been determined. However, since the ER-located ethylene receptors (ETRs) are largely dependent on Cu supply by RAN1, an ER location has been proposed [42–44]. Thus, unlike storage organelles as chloroplasts, mitochondria or vacuoles, the ER could act as a more reliable indicator of the steady-state Cu availability in the cell.

Notably, several studies have recently reported a central role of the ER in sensing/transducing cellular stresses [37,45]. In an attempt to identify ER perturbations that activate SPL7, our initial data suggest an impact in the ER protein-folding capacity during Cu starvation and how ER stress treatments slightly induced selected SPL7-targets in mild Cu-sufficient seedlings. Whether the initial tethering of SPL7 to the ER-membrane could be a cellular strategy to sense Cu limitation through the stress it imposes to the ER needs to be further investigated. Within this context, it is also worth mentioning that the growth inhibitory effect of fumonisin B1 (FB1) was found attenuated in the *fbr6* mutant, representing the SPL7-related SPL14 TF [2]. The apoptotic effect of the mycotoxin FB1 is related to a reduction in the cellular ceramide levels, a likely signal for ER-stress [46–48]. Given the conservation of the putative TMD among the large SPLs including SPL14, it would also be interesting to address if the strategy proposed for SPL7 could be extended to this class of TFs.

On the other hand, given that the SPL7 orthologs in single-celled algae lack a TMD, this domain could represent an innovation in the evolution of land plants [16]. The positive selection of the TMD may be related to the multicellular and more complex nature of land plants, where many different cell types likely differ in their requirements for Cu and their demand probably even changes with growth and development. Thus, anchoring SPL7-like proteins to membranes could play a role in fine-tuning their activities in a more cell-autonomous context. However, although further comparative studies between Crr1 and SPL7-like proteins are required to provide a more thorough answer, the existence of additional regulatory levels for these TFs in higher plants seems likely.

Based on our data, we also propose SPL7 homodimerization as another checkpoint in the regulation of SPL7 activity. Indeed, independent *in vivo* approaches indicated that the SPL7 N-terminal half is prone to self-dimerization. Accordingly, only SPL7 protein fragments encompassing at least the conserved signature RXSXKLX_4_PX_3_PX_2_LX_7_LX_7_EX_3_RXGCX_3_T denoted the IRPGC domain (albeit extended compared to previous reports [16,17]), were isolated in a Y2H screen using SPL7 as bait. Consequently, this signature could be considered to represent a dimerization domain. Homodimer formation involving this domain in the N-terminal half of SPL7 would also explain our observations on co-immunoprecipitated N-terminal SPL7 fragments, most likely generated through post-translational processing as discussed above. Similarly, only split YFP fragments fused as N-terminal tags to SPL7 were successful in BiFC assays. Furthermore, the reconstituted YFP fluorophore signal for N-terminal fusions illuminated the nuclear surroundings and cytoplasmic filaments, in an ER-like distribution. However, these results seem to contradict observations on GFP-tagged SPL7-like proteins clearly located in the nucleus when overexpressed in heterologous systems (our results and [14,26]). Therefore, it is tempting to speculate that SPL7 preferentially enters the nucleus as a monomer. Exclusion of the dimer may be the result of the large size of the protein complex formed or of masking the NLS (Figure 8). In addition, rapid degradation or instability of SPL7 dimers cannot be ruled out as GFP::SPL7 was not easily detectable outside the nucleus neither in fluorescence microscopy nor in biochemical approaches. Hence dimerization, likely promoted by increasing amounts of released SPL7 protein, may be part of a negative feedback mechanism to attenuate the homeostatic Cu deficiency response and eventually avoid spurious effects. Interestingly, given the conservation of the IRPGC signature not only in SPL7 orthologous proteins but also in closely related large SPLs in Arabidopsis [16], homodimerization, or even heterodimerization, may represent a more general regulatory feature of this type of SBP-domain TFs.

The participation of additional SPL7-interacting proteins in the SPL7 post-translational regulation mechanism cannot be excluded (Figure 8). Indeed, KIN17 associates with SPL7 in order to stimulate SPL7-targets and counteract the oxidative stress under Cu deprivation [26]. Nevertheless, we are not aware of mutants for other genes with a similar or even close impact on the global response to Cu deficiency as *spl7* mutants have. Therefore we assume that the likely SPL7-interactome consists of largely functionally redundant components that probably contribute more to refine SPL7 function, rather than to its activation.

Importantly, SPL7 is expected to undergo a high turnover because different tagged full-sized SPL7-like proteins could not be clearly detected in stable transgenics, despite their functionality (our observations and [9,10,14]) and reasonable transgene transcript levels (Additional file 5: Figure S5). We also did not succeed to trace SPL7 *in planta* by observing different tissues at different time-points or using different tags, growth conditions or protein degradation inhibitors (data not shown). However, we demonstrated that expression of an N-terminal GFP-tagged SPL7 fragment including the SBP-domain but lacking the downstream IRPGC domain could be detected and resulted in a constitutive SPL7 function-related response irrespective of the Cu availability. A similar behaviour has been reported for other ER-attached proteins. A constitutive ethylene triple response is achieved by expressing putative C-terminal EIN2-cleaved fragments [45]. Similarly, the *anac017*-2 mutant, rendering a truncated version of ANAC017 without the TMD, induces its target *ALTERNATIVE OXIDASE1* (AOX1), even in non-H_2_O_2_-treated plants [37]. Remarkably, the constitutive transcriptional activity of SPL7 targets in SPL7-SBP transgenic lines, even during non-physiological Cu excess, seem to contradict previous data showing that Cu ions negatively interfere the DNA-binding capacity of the SPL7 SBP-domain *in vitro* [14]. One should take into account that Cu ions cannot move freely within cells due to the efficient Cu-chelating capacity of cells [49]. Moreover, a direct interaction between SPL7 and free Cu ions seems unlikely because Cu is mostly stored in organelles like the chloroplasts and mitochondria, whereas SPL7 distributes between endomembranes and nucleus. Nevertheless, a slight decrease in SPL7-targets could be even noticed in the SPL7-SBP plants during the transition from Cu deficiency to sufficiency. We, therefore, propose that the effect of Cu ions on the functionality of SPL7 is mediated by some interacting factor(s), such as specific metallochaperones (Figure 8). The respective interacting SPL7 domain(s) is then most likely C-terminal of the SBP-domain. An overlap with the IRPGC domain, as the main conserved signature within this region, cannot be excluded. Whether the dimerization through this domain constitutes a possible regulatory mechanism promoting SPL7 turnover needs to be further addressed.

## Conclusions

Altogether, our data provide novel insights into the molecular mechanisms underlying the role of the SPL7 TF in orchestrating Cu homeostasis in plants. Additionally, the mechanism of action we have reported here for SPL7 may possibly be extrapolated to other large SBP-domain proteins because a conservation of particular structural features is suggested on the basis of amino acid sequence similarities.

## Methods

### Plant growth and manipulation

The wild-type line used in all the experiments corresponded to the *Arabidopsis thaliana* ecotype Columbia (Col-0). The *spl7-*2 mutant has been previously described by Bernal and colleagues [9]. Seeds were stratified at 4°C for 2 days prior to be sown. For *in vitro* culture, seeds were surface sterilized with sequential washes in ethanol 70% (5 min), bleach (5 min), water (2× 2 min), resuspended in agar 0.1% (w/v) and sown on half-strength MS medium plates (½ MS; Sigma) supplemented with sucrose 1% (w/v) and CuSO_4_ as indicated. Cu-deficient growth conditions were achieved by adding the specific Cu chelator bathocuproine disulphonate (BCS; Sigma-Aldrich) to the medium. In all cases, long day conditions (16 h light, 20-23°C/8 h darkness, 16°C) were applied. To generate stable transgenic lines, constructs were introduced in wild-type and *spl7*-2 mutant plants using *Agrobacterium tumefaciens* GV3101 (pMP90RK) in the floral-dip method [50,51].

### Constructs

cDNA fragments corresponding to the entire coding sequence or selected regions of *SPL7* (AT5G18830.1) were amplified with specific oligonucleotides (Additional file 6: Table S1) and cloned into *pDONR207* by means of the Gateway BP clonase II (Invitrogen). The generated entry clones were further recombined into the *pMDC43* or *pMDC201* vectors with LR clonase II (Invitrogen) to add a GFP tag at either the amino- or carboxi-terminus, respectively [52]. Similarly, the *pALLIGATOR2* vector was chosen to add a 3xHA tag to the N-terminus of full-sized SPL7 (HA::SPL7) [53]. For BiFC, full-sized *SPL7* in *pDONR207* was LR-recombined into both the *pYFN43* and *pYFC43* destiny vectors providing N-terminal the two halves of YFP [54]. The ER marker fused to mCherry (ER-rk) used for subcellular co-localizations was described in Nelson and colleagues [28].

### Y2H screen

The Y2H assay was performed by Hybrigenics Services SAS using a fragment of SPL7 (aa residues 133 to 762) as bait to screen a random-primed cDNA prey library prepared from 1-week-old Arabidopsis seedlings.

### Subcellular localization and bimolecular fluorescence complementation assay on tobacco leaves

To determine the subcellular localization of truncated SPL7 protein versions, *Nicotiana benthamiana* (tobacco) young leaves were co-infiltrated with diluted cultures of *A. tumefaciens* harbouring CaMV 35S promoter-driven transgene constructs expressing fluorescent-tagged proteins of interest together with the *p19* plasmid [55] in infiltration buffer [D-glucose 0.5% (w/v); MES 10 mM; MgCl_2_ 10 mM; acetosyringone 0.1 mM] at an OD_600_ 0.2. Small pieces of leaves were excised 2–3 days after infiltration and examined in a confocal laser scanning microscope (Zeiss LSM700) using filters to select for the GFP and chlorophyll signal. For BiFC assays, the *pYFN43::SPL7* (nYFP::SPL7) and *pYFC43::SPL7* (cYFP::SPL7) destiny vectors were individually or co-expressed in leaf epidermal tobacco cells as indicated above, and examined using widefield epifluorescence microscopy (Olympus BX61) and confocal laser scanning microscopy to asses the restoration of the YFP signal.

### Biochemical fractionation

Biochemical fractionation was carried out according to Sáez and collaborators [56] with modifications: 3 g of transiently transformed tobacco leaves were homogenised in 3 volumes of extraction buffer [Tris–HCl 20 mM pH 7.4; glycerol 25% (v/v); KCl 20 mM; MgCl_2_ 2.5 mM; EDTA 2 mM; sucrose 250 mM; Pefabloc 1 mM; cOmplete Protease Inhibitor Cocktail (Roche) 1X], filtered through 2 Miracloth layers and centrifuged at 1000 g for 10 min at 4°C to pellet nuclei. Pellets were gently rinsed with 2 mL of Nuclei Wash Buffer [Tris–HCl 20 mM pH 7.4; glycerol 25% (v/v); MgCl_2_ 2.5 mM; Triton X-100 0.5% (v/v)]. After centrifugation at 1000 g for 30 s pellets were resuspended in 5 volumes of Medium Salt Buffer [Tris–HCl 20 mM pH 7.4; glycerol 5%; NaCl 0.4 M; β-mercaptoethanol 1 mM; EDTA 1 mM; Pefabloc 0.5 mM; cOmplete Protease Inhibitor Cocktail 1X and stored frozen. Samples were thawed on ice, stirred for 15 min and centrifuged at 10000 g for 10 min to recover the supernatant, which was considered as the nuclear enriched fraction. To obtain the microsomal fraction, the initial supernatant was submitted to ultracentrifugation at 100000 g for 1 h in a SW-44 Ti rotor (Beckman) and the sediment was resuspended in extraction buffer. The remaining supernatant was used as the cytosolic fraction. All fractions were concentrated by means of Microcon Centrifugal Filter Devices (Merck Millipore) columns and equal amounts of proteins loaded on 10% NuPAGE precasted gels (Life Technologies). Antibodies used for Western blot were: anti-GFP (1:1000; clones 7.1 and 13.1 Roche), anti-TPR7 (1:1000 [57]), anti-PEPC (1:15000; Rockland) and anti-H3 (1:10000; Abcam).

### Induction of ER stress in Arabidopsis seedlings

ER stress was induced as described by Li and colleagues [32]. Thereto, wild-type and *spl7*-2 seedlings were grown on ½ MS supplemented with sucrose 1% (w/v) and CuSO_4_ 0.5 μM for 5 days. Subsequently, they were cultured in liquid ½ MS treated with tunicamycin (5 μg/mL) or DTT (2 mM) during 3 h with gentle shaking. Material was harvested and used for gene expression assays by qPCR.

### Gene expression analysis by quantitative real-time PCR

For gene expression assays, total RNA was prepared with the Spectrum Plant Total RNA Kit (Sigma-Aldrich) according to manufacturer’s instructions. The RNA integrity was visualized in ethidium bromide-agarose gels. RNA samples were treated with DNase I recombinant RNase-free (Roche) and reverse transcribed to cDNA with the Superscript II Reverse Transcriptase (Invitrogen). qPCR analysis were carried out in an iQ5 Real-Time PCR Detection System (Bio-Rad) with EvaGreen (Biotium) and specific primers (see Additional file 7: Table S2) using an initial cycle at 95°C for 3 min and 40 cycles consisting in 95°C for 10 s, 58°C for 20 s and 72°C for 20 s. *ACT2* and *EF1* were used to normalize gene expression values. Statistical analysis of at least three independent biological samples was performed using Excel (Microsoft Corporation). Student’s *t*-test was used to determine statistically significant differences with a p < 0.05 or p < 0.01 as level of significance.

### Co-immunoprecipitation

Total protein extracts from tobacco leaves transiently co-expressing HA::SPL7 and GFP::SPL7 were prepared by grinding frozen material in co-immunoprecipitation (CoIP) buffer [PIPES-KOH 10 mM pH 7; NaCl 50 mM; EDTA 0.5 mM pH 8.0; Triton X-100 0.5% (v/v); cOmplete Protease Inhibitor Cocktail 1X] and crosslinked to a limited extent with formaldehyde 1% (v/v). Samples were centrifuged at maximum speed at 4°C for 5 min and 0.4 volumes 2 M glycine were added to the supernatants in order to stop the crosslinking. Next, HA::SPL7 was pulled-down by incubating 1 mL of protein extract with 1.5 μg of anti-HA high affinity antibody (3 F10 clone, Roche) on rotation at 4°C for 2 h. Then, 50 μL of equilibrated Protein G Mag Sepharose Xtra (GE Healthcare) magnetic beads were added to extracts and rotated overnight at 4°C. Finally, beads were recovered, washed three times with 1 mL CoIP Buffer supplemented with NaCl 300 mM, Triton X-100 0.1% (v/v) and cOmplete Protease Inhibitor Cocktail 1X and boiled at 95°C for 10 min with 50 μL SDS-PAGE load buffer 4X. GFP::SPL7 co-immunopreciptitation was assessed by Western blot with anti-GFP-HRP (1:1000; Molecular Probes) and anti-HA-HRP (1:1000; Roche) antibodies.

### Availability of supporting data

All the supporting data are included as additional files.

## Additional files

Additional file 1: Figure S1. Multiple sequence alignment of carboxy-termini and intermediate regions of SPL7 orthologous proteins. (a) and (b) Amino acid sequences from SPL7-like proteins were aligned using ClustalW within the MacVector software package with default parameters. Conserved signatures are indicated with red squares: TMD in (a) and IRPGC putative dimerization domain in (b). (c) Sequence logo IRPGC putative dimerization domain obtained with the Weblogo interface (http://weblogo.berkeley.edu/logo.cgi). Genbank accession numbers: *Chlamydomonas reinhardtii* AAY33924; *Chlorella variabilis* XP_005851140.1; *Volvox carteri* XP_002948544.1; *Arabidopsis thaliana* At5g18830.1; *Arabidopsis lyrata* XP_002871844.1; *Capsella rubella* EOA22312.1*; Thellungiella halophila* BAJ34638.1; *Sorghum bicolor* XP_002439790.1; *Oryza sativa* NP_001055522.1; *Amborella trichopoda* ERN18478; *Vitis vinifera* XP_002277039.1; *Physcomitrella patens* ABM67299.1; *Hordeum vulgare* BAJ96319.1; *Ricinus communis* XP_002516839.1; *Glycine max* XP_003547221.1; *Picea sitchensis* ABR17971.1; *Solanum lycopersicum* XP_004229492.1; *Theobroma cacao* EOY06351.1; *Zea mays* AFW81967.1. Additional file 2: Figure S2. Nuclear localization of the N-terminal GFP tagged SPL7. Epidermal tobacco leaves were co-infiltrated with GFP::SPL7 and the nuclear marker pSAT6-mCherry-VirD2NLS [58] and examined using confocal microscopy. A representative image of the respective GFP and mCherry signals are shown together with the corresponding bright field and merged images. Scale bar, 10 μm. Additional file 3: Figure S3. A yeast two-hybrid assay uncovers SPL7 homodimerization. Using a SPL7 bait including aa residues 133 to 762 in a Y2H assay retrieved 8 independent preys corresponding to SPL7 derived polypeptides. The cartoon depicts their alignment relative to the bait and the full-size SPL7 protein with the conserved domains (SBP; IRPGC; TMD) indicated with squares. The common region shared by all preys (shaded green) and the presence of the IRPGC domain (shaded red) are highlighted. The position of the N- and C-terminal amino acid residues relative to the full-size SPL7 protein is provided. Additional file 4: Figure S4. Complementation of the *spl7-*2 mutant phenotype by two different SPL7 protein derived polypeptides. (a) Seedlings of the wild-type (WT), *spl7*-2 mutant and transformed *spl7*-2 lines expressing the indicated GFP-tagged SPL7 protein-derived polypeptides (GFP-SPL7; GFP-SBP) grown on vertically placed agar plates containing ½ MS supplemented with sucrose 1% and BCS 50 μM (−Cu) or CuSO_4_ 5 μM (+Cu) for 5 days before imaging (upper panel). (b) Root length measurement of plants in (a). Bars represent the mean with error bars corresponding to the standard deviation (n > 9). Asterisk indicates statistically significant difference to comparably grown wild type according to Student’s *t*-test (p < 0.01). (c) Complementation of *spl7*-2 phenotypes in adult plants. Phenotypes of 1-month-old *spl7*-2 mutant plants complemented with the respective SPL7 protein derived polypeptides in comparison to wild-type and *spl7*-2 single mutant plants grown on standard soil. Additional file 5: Figure S5. Detection of endogenous and transgene derived *SPL7* transcripts in GFP::SPL7 transgenic lines. Total RNA was isolated from 7-day-old seedlings grown under Cu deficiency (BCS 50 μM) and Cu mild excess (Cu 5 μM) and corresponding to the WT, *spl7*-2 mutant and two transgenic lines expressing *GFP-SPL7* against an *spl7*-2 mutant background. Relative transcript levels of endogenous *SPL7* (with an *SPL7* specific primer pair) and transgenic *GFP-SPL7* (with a *GFP* specific primer pair) were determined by qPCR. Error bars represent the standard deviation of 3 technical replicates. Additional file 6: Table S1. Oligonucleotides used for cloning. The name, sequence (5′-3′) and target for each oligonucleotide is provided. Additional file 7: Table S2. Oligonucleotides used for quantitative real-time PCR. The name and sequence (5′-3′) for each combination of oligonucleotides are provided.
