# Supplementary material for: Functional characterisation of Arabidopsis SPL7 conserved protein domains suggests novel regulatory mechanisms in the Cu deficiency response
Source: BMC Plant Biol. 2014 Aug 30;14:231. doi: 10.1186/s12870-014-0231-5 (PMC4158090; doi:10.1186/s12870-014-0231-5)
Supplement: Additional file 1: Figure S1. — Multiple sequence alignment of carboxy-termini and intermediate regions of SPL7 orthologous proteins. (a) and (b) Amino acid sequences from SPL7-like proteins were aligned using ClustalW within the MacVector software package with default parameters. Conserved signatures are indicated with red squares: TMD in (a) and IRPGC putative dimerization domain in (b). (c) Sequence logo IRPGC putative dimerization domain obtained with the Weblogo interface (http://weblogo.berkeley.edu/logo.cgi). Genbank accession numbers: Chlamydomonas reinhardtii AAY33924; Chlorella variabilis XP_005851140.1; Volvox carteri XP_002948544.1; Arabidopsis thaliana At5g18830.1; Arabidopsis lyrata XP_002871844.1; Capsella rubella EOA22312.1; Thellungiella halophila BAJ34638.1; Sorghum bicolor XP_002439790.1; Oryza sativa NP_001055522.1; Amborella trichopoda ERN18478; Vitis vinifera XP_002277039.1; Physcomitrella patens ABM67299.1; Hordeum vulgare BAJ96319.1; Ricinus communis XP_002516839.1; Glycine max XP_003547221.1; Picea sitchensis ABR17971.1; Solanum lycopersicum XP_004229492.1; Theobroma cacao EOY06351.1; Zea mays AFW81967.1. [file 12870_2014_231_MOESM1_ESM.docx]

**
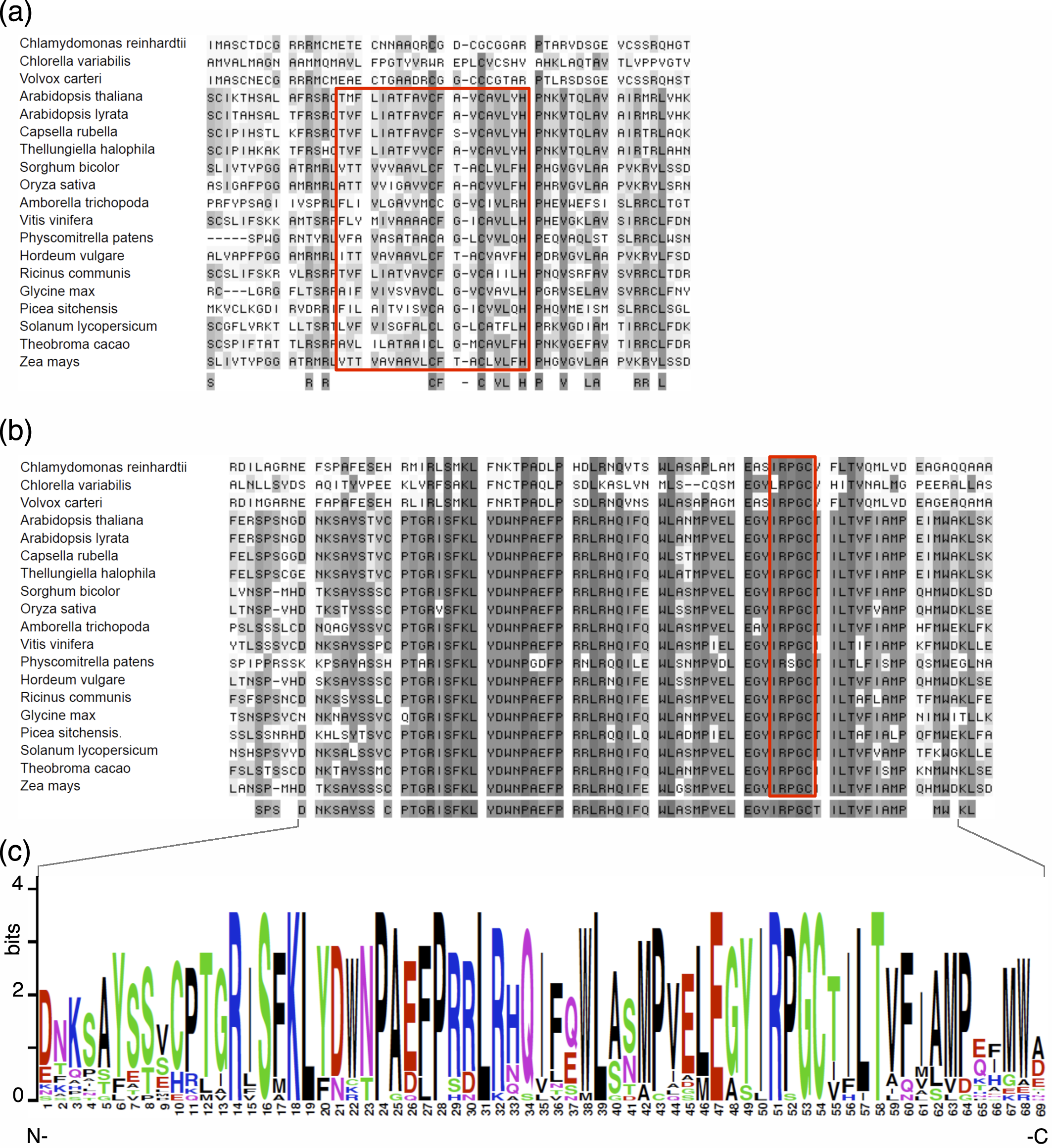
**

**Additional file 1: Figure S1.** Multiple sequence alignment of carboxy-termini and intermediate regions of SPL7 orthologous proteins. (a) and (b) Amino acid sequences from SPL7-like proteins were aligned using ClustalW within the MacVector software package with default parameters. Conserved signatures are indicated with red squares: TMD in (a) and IRPGC putative dimerization domain in (b). (c) Sequence logo IRPGC putative dimerization domain obtained with the Weblogo interface (http://weblogo.berkeley.edu/logo.cgi). Genbank accession numbers: *Chlamydomonas reinhardtii* AAY33924; *Chlorella variabilis* XP_005851140.1; *Volvox carteri* XP_002948544.1; *Arabidopsis thaliana* At5g18830.1; *Arabidopsis lyrata* XP_002871844.1; *Capsella rubella* EOA22312.1*; Thellungiella halophila* BAJ34638.1; *Sorghum bicolor* XP_002439790.1; *Oryza sativa* NP_001055522.1; *Amborella trichopoda* ERN18478; *Vitis vinifera* XP_002277039.1; *Physcomitrella patens* ABM67299.1; *Hordeum vulgare* BAJ96319.1; *Ricinus communis* XP_002516839.1; *Glycine max* XP_003547221.1; *Picea sitchensis* ABR17971.1; *Solanum lycopersicum* XP_004229492.1; *Theobroma cacao* EOY06351.1; *Zea mays* AFW81967.1
