# Supplementary material for: Functional characterisation of Arabidopsis SPL7 conserved protein domains suggests novel regulatory mechanisms in the Cu deficiency response
Source: BMC Plant Biol. 2014 Aug 30;14:231. doi: 10.1186/s12870-014-0231-5 (PMC4158090; doi:10.1186/s12870-014-0231-5)
Supplement: Additional file 2: Figure S2. — Nuclear localization of the N-terminal GFP tagged SPL7. Epidermal tobacco leaves were co-infiltrated with GFP::SPL7 and the nuclear marker pSAT6-mCherry-VirD2NLS [58] and examined using confocal microscopy. A representative image of the respective GFP and mCherry signals are shown together with the corresponding bright field and merged images. Scale bar, 10 μm. [file 12870_2014_231_MOESM2_ESM.doc]

**
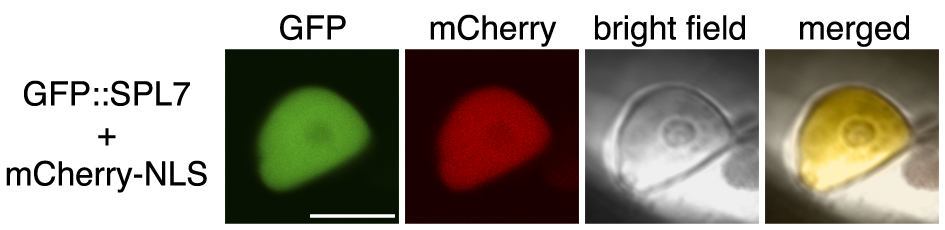
**

**Additional file 2: Figure S2.** Nuclear localization of the N-terminal GFP-tagged SPL7. Tobacco leaves were co-infiltrated with GFP::SPL7 and the nuclear marker pSAT6-mCherry-VirD2NLS [58] and examined in epidermal cells using confocal microscopy. A representative image of the respective GFP and mCherry signals are shown together with the corresponding bright field and merged images. Scale bar, 10 µm.
