# Supplementary material for: Functional characterisation of Arabidopsis SPL7 conserved protein domains suggests novel regulatory mechanisms in the Cu deficiency response
Source: BMC Plant Biol. 2014 Aug 30;14:231. doi: 10.1186/s12870-014-0231-5 (PMC4158090; doi:10.1186/s12870-014-0231-5)
Supplement: Additional file 3: Figure S3. — A yeast two-hybrid assay uncovers SPL7 homodimerization. Using a SPL7 bait including aa residues 133 to 762 in a Y2H assay retrieved 8 independent preys corresponding to SPL7 derived polypeptides. The cartoon depicts their alignment relative to the bait and the full-size SPL7 protein with the conserved domains (SBP; IRPGC; TMD) indicated with squares. The common region shared by all preys (shaded green) and the presence of the IRPGC domain (shaded red) are highlighted. The position of the N- and C-terminal amino acid residues relative to the full-size SPL7 protein is provided. [file 12870_2014_231_MOESM3_ESM.docx]

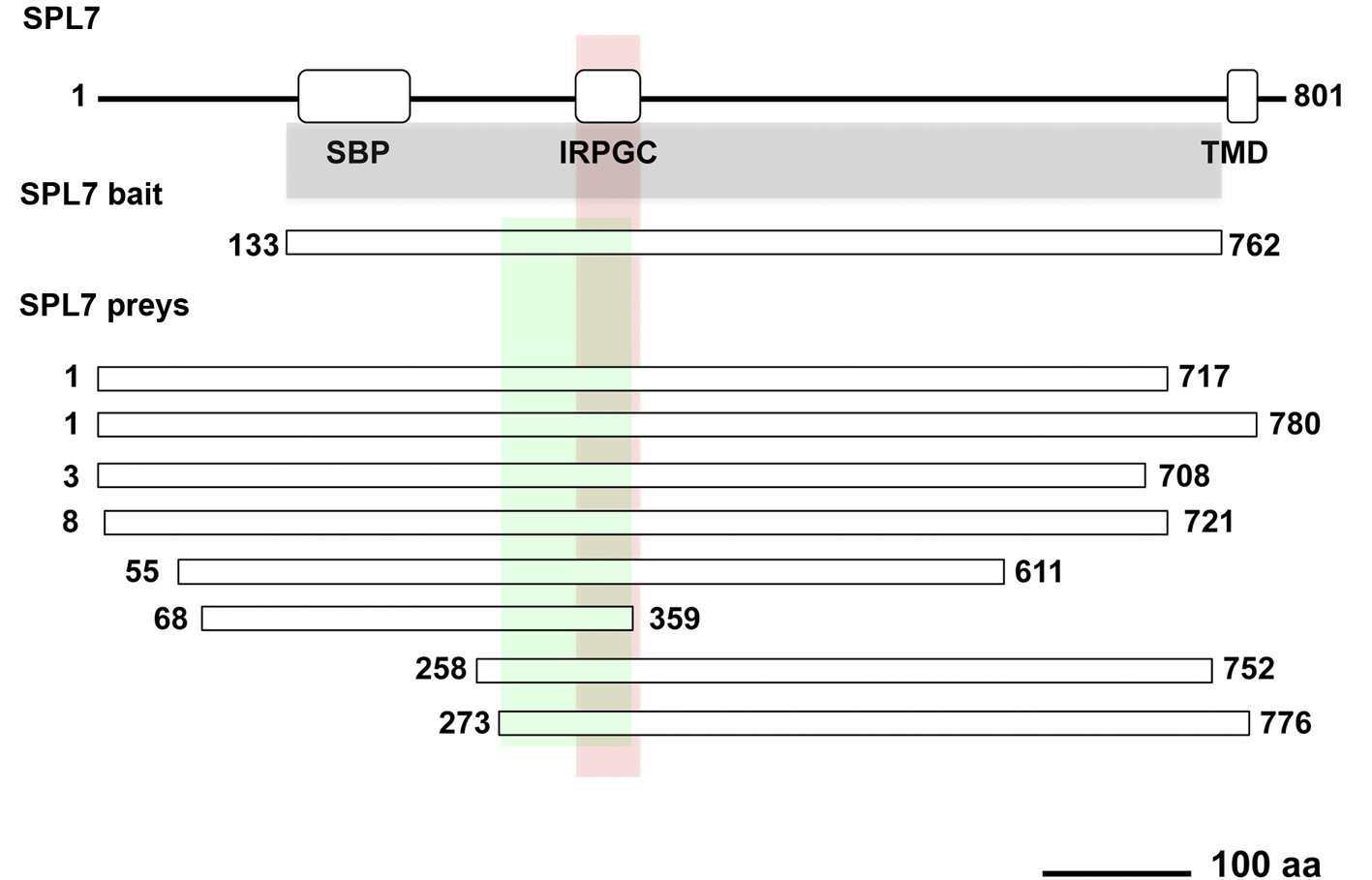


**Additional file 3: Figure S3.** A yeast two-hybrid assay uncovers SPL7 homodimerization. Using a SPL7 bait including aa residues 133 to 762 in a Y2H assay retrieved 8 independent preys corresponding to SPL7 derived polypeptides. The cartoon depicts their alignment relative to the bait and the full-size SPL7 protein with the conserved domains (SBP; IRPGC; TMD) indicated with squares. The common region shared by all preys (shaded green) and the presence of the IRPGC domain (shaded red) are highlighted. The position of the N- and C-terminal amino acid residues relative to the full-size SPL7 protein is provided.
