# Supplementary material for: Functional characterisation of Arabidopsis SPL7 conserved protein domains suggests novel regulatory mechanisms in the Cu deficiency response
Source: BMC Plant Biol. 2014 Aug 30;14:231. doi: 10.1186/s12870-014-0231-5 (PMC4158090; doi:10.1186/s12870-014-0231-5)
Supplement: Additional file 4: Figure S4. — Complementation of the spl7-2 mutant phenotype by two different SPL7 protein derived polypeptides. (a) Seedlings of the wild-type (WT), spl7-2 mutant and transformed spl7-2 lines expressing the indicated GFP-tagged SPL7 protein-derived polypeptides (GFP-SPL7; GFP-SBP) grown on vertically placed agar plates containing ½ MS supplemented with sucrose 1% and BCS 50 μM (−Cu) or CuSO4 5 μM (+Cu) for 5 days before imaging (upper panel). (b) Root length measurement of plants in (a). Bars represent the mean with error bars corresponding to the standard deviation (n > 9). Asterisk indicates statistically significant difference to comparably grown wild type according to Student’s t-test (p < 0.01). (c) Complementation of spl7-2 phenotypes in adult plants. Phenotypes of 1-month-old spl7-2 mutant plants complemented with the respective SPL7 protein derived polypeptides in comparison to wild-type and spl7-2 single mutant plants grown on standard soil. [file 12870_2014_231_MOESM4_ESM.docx]

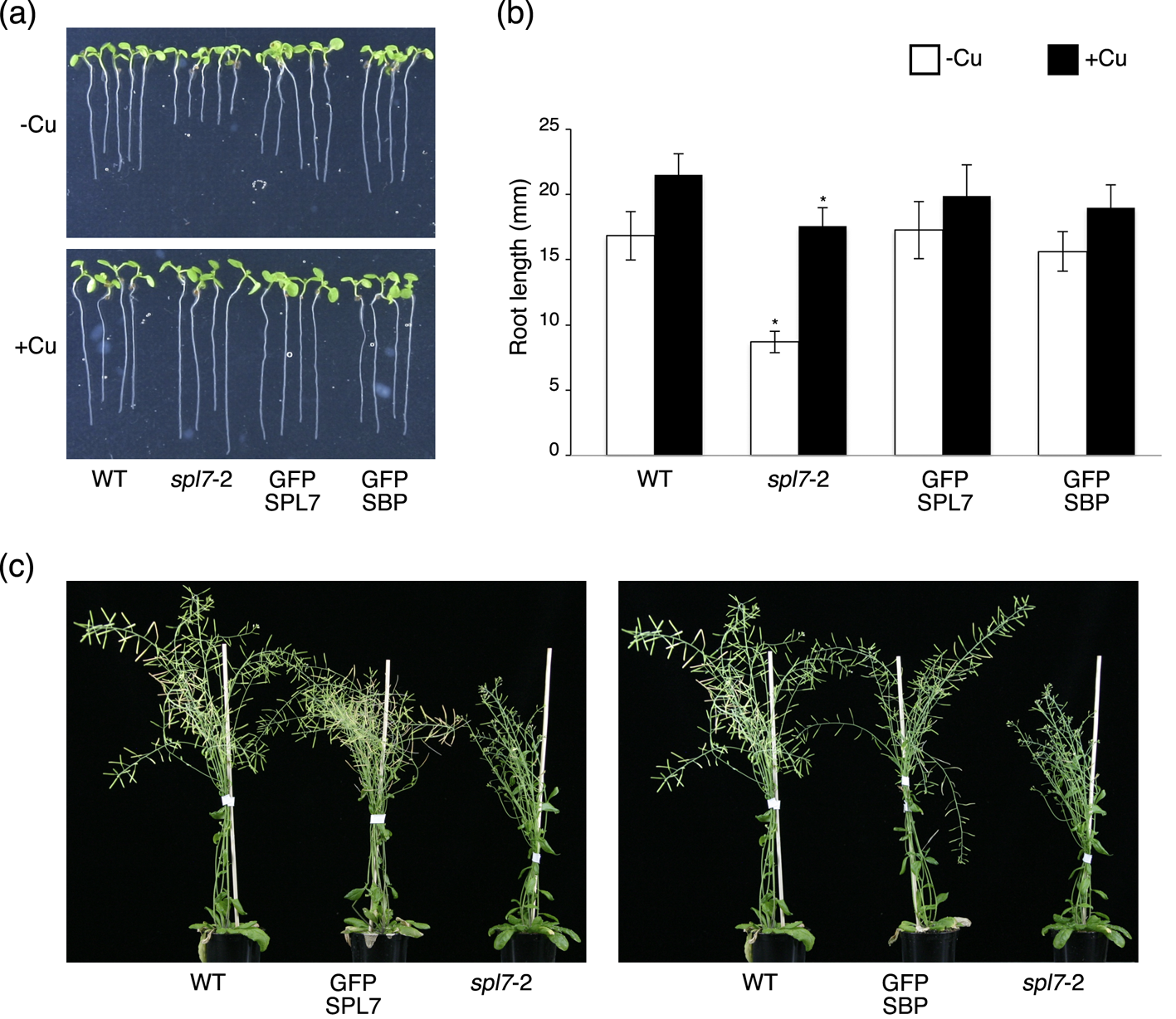


**Additional file 4: Figure S4.** Complementation of the *spl7-*2 mutant phenotype by two different SPL7 protein derived polypeptides. (a) Seedlings of the wild-type (WT), *spl7*-2 mutant and transformed *spl7*-2 lines expressing the indicated GFP-tagged SPL7 protein-derived polypeptides (GFP-SPL7; GFP-SBP) grown on vertically placed agar plates containing ½ MS supplemented with sucrose 1% and BCS 50 µM (-Cu) or CuSO_4_ 5 µM (+Cu) for 5 days before imaging (upper panel). (b) Root length measurement of plants in (a). Bars represent the mean with error bars corresponding to the standard deviation (n > 9). Asterisk indicates statistically significant difference to comparably grown wild type according to Student’s *t*-test (p<0.01). (c) Complementation of *spl7*-2 phenotypes in adult plants. Phenotypes of 1-month-old *spl7*-2 mutant plants complemented with the respective SPL7 protein derived polypeptides in comparison to wild-type and *spl7*-2 single mutant plants grown on standard soil.
