# Supplementary material for: Functional characterisation of Arabidopsis SPL7 conserved protein domains suggests novel regulatory mechanisms in the Cu deficiency response
Source: BMC Plant Biol. 2014 Aug 30;14:231. doi: 10.1186/s12870-014-0231-5 (PMC4158090; doi:10.1186/s12870-014-0231-5)
Supplement: Additional file 5: Figure S5. — Detection of endogenous and transgene derived SPL7 transcripts in GFP::SPL7 transgenic lines. Total RNA was isolated from 7-day-old seedlings grown under Cu deficiency (BCS 50 μM) and Cu mild excess (Cu 5 μM) and corresponding to the WT, spl7-2 mutant and two transgenic lines expressing GFP-SPL7 against an spl7-2 mutant background. Relative transcript levels of endogenous SPL7 (with an SPL7 specific primer pair) and transgenic GFP-SPL7 (with a GFP specific primer pair) were determined by qPCR. Error bars represent the standard deviation of 3 technical replicates. [file 12870_2014_231_MOESM5_ESM.docx]

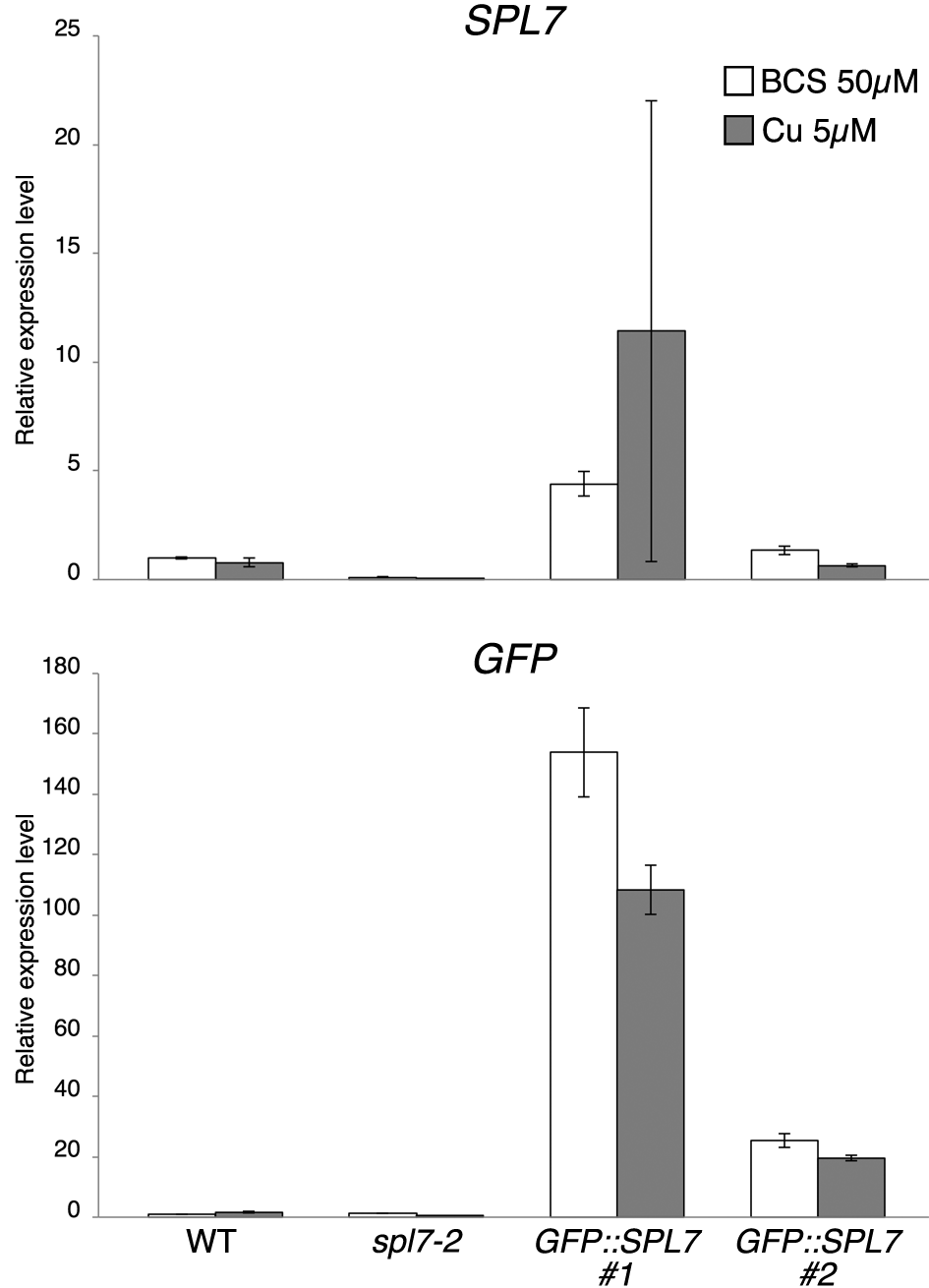


**Additional file 5: Figure S5.** Detection of endogenous and transgene derived *SPL7* transcripts in GFP::SPL7 transgenic lines. Total RNA was isolated from 7-day-old seedlings grown under Cu deficiency (BCS 50 µM) and Cu mild excess (Cu 5 µM) and corresponding to the wild type (WT), *spl7*-2 mutant and two transgenic lines expressing *GFP-SPL7* against an *spl7*-2 mutant background. Relative transcript levels of endogenous *SPL7* (with an *SPL7* specific primer pair) and transgenic *GFP-SPL7* (with a *GFP* specific primer pair) were determined by qPCR. Error bars represent the standard deviation of 3 technical replicates.
