# Supplementary material for: Functional characterisation of Arabidopsis SPL7 conserved protein domains suggests novel regulatory mechanisms in the Cu deficiency response
Source: BMC Plant Biol. 2014 Aug 30;14:231. doi: 10.1186/s12870-014-0231-5 (PMC4158090; doi:10.1186/s12870-014-0231-5)
Supplement: Additional file 6: Table S1. — Oligonucleotides used for cloning. The name, sequence (5′-3′) and target for each oligonucleotide is provided. [file 12870_2014_231_MOESM6_ESM.doc]

**Additional file 6: Table S1. Oligonucleotides used for cloning.** The name, sequence (5’-3’) and target for each oligonucleotide is provided.

| **NAME** | **SEQUENCE 5'-3'** | **USE** |
| --- | --- | --- |
| **SPL7-ATG-F** | GGGGACAAGTTTGTACAAAAAAGCAGGCTCTTCTCTGTCGCAATCGCCACCAC | Cloning full-sized SPL7 cDNA |
| **SPL7-TAG-R** | GGGGGACCACTTTGTACAAGAAAGCTGGGTAAATTTTGTGTACCAATCTCATTCG |
| **SPL7-ATG-F** | GGGGACAAGTTTGTACAAAAAAGCAGGCTCTTCTCTGTCGCAATCGCCACCAC | Cloning full-sized SPL7 cDNA without STOP |
| **SPL7-nonSTOP-R** | GGGGACCACTTTGTACAAGAAAGCTGGGTCAATTTTGTGTACCAATCTCATT |
| **SPL7-ATG-F** | GGGGACAAGTTTGTACAAAAAAGCAGGCTCTTCTCTGTCGCAATCGCCACCAC | Cloning full-sized SPL7 cDNA |
| **SPL7-SBP-R** | GGGGGACCACTTTGTACAAGAAAGCTGGGTCCATCCTCAACATCAATGACGCTG |
| **SPL7-TMD-F** | GGGGACAAGTTTGTACAAAAAAGCAGGCTGGCCAAGGAAGTCGTGTATAAAAACG | Cloning SPL7 cDNA  (746-801 aa) |
| **SPL7-TAG-R** | GGGGGACCACTTTGTACAAGAAAGCTGGGTAAATTTTGTGTACCAATCTCATTCG |
