# Supplementary material for: Functional characterisation of Arabidopsis SPL7 conserved protein domains suggests novel regulatory mechanisms in the Cu deficiency response
Source: BMC Plant Biol. 2014 Aug 30;14:231. doi: 10.1186/s12870-014-0231-5 (PMC4158090; doi:10.1186/s12870-014-0231-5)
Supplement: Additional file 7: Table S2. — Oligonucleotides used for quantitative real-time PCR. The name and sequence (5′-3′) for each combination of oligonucleotides are provided. [file 12870_2014_231_MOESM7_ESM.doc]

**Additional file 7: Table S2. Oligonucleotides used for quantitative real-time PCR.** The name and sequence (5’-3’) for each combination of oligonucleotides are provided.

| **GENE** | **SEQUENCE 5'-3'** | **EFFICIENCY** |
| --- | --- | --- |
| ***ACT2*** | CTTGCACCAAGCAGCATGAA / CCGATCCAGACACTGTACTTCCTT | 86% |
| ***BiP1,2*** | TCAGTCCTGAGGAGATTAGTGCT / TGCCTTTGAGCATCATTGAA | 82.7% |
| ***CCH*** | GTGGAGGCTGAAGCTGAGCCAA / TCGGCTTCTGCGGCTTTTGGT | 108.4% |
| ***COPT1*** | CGAATGGCTTGCTCATTCCT / GGTATACACGGCGGTTTGGAT | 97.7% |
| ***COPT2*** | CGCCGGCTATGGCGTTGGTT / GCAGCCTGAAGACGGCGGAA | 93.6% |
| ***CRT1*** | AGACCTTAGTCTTCCAATTCTC / CCATTGTAAGTAAGGATAGCATG | 99.4% |
| ***EF1*** | TGGTGACGCTGGTATGGTTA / TCCTTCTTGTCCACGCTCTT | 91.8% |
| ***FSD1*** | GGATTCACAGCATTGGGAGT / TGGAGGAAAACCATCAGGAG | 93.6% |
| ***GFP*** | CTGTTCCTTGGCCAACACTT / ATCCTGTTGACGAGGGTGTC | 99.9% |
| ***PDIL*** | CTCGTGAAGCTGAGGGTATTG / TGTGCGAAATCTAACTCAGAG | 76.5% |
| ***SPL7*** | CAGGCAGACTGTTCACCAGA / AGTTTGACGGGACCTGAATG | 99.0% |
| ***MIR398C*** | CAATCAACGGCTATAACGACGCTACG / GTGACCTGAGAACACATGAAACGAGAG | 99.9% |
| ***bZIP60*** | CGATGATGCTGTGGCTAAAA / TCTCAAGCATTCTCTTTCGAGAT | 97.3% |
